# Anti-correlation time-frequency synergy and dual-granularity fusion for sleep stage classification

**DOI:** 10.3389/fnins.2026.1944816

**Published:** 2026-10-20

**Authors:** Jie Pan, Xingxing Zhao, Xiaoyu Zou, Pengjun Zhao, Leilei Wang, Chengcheng Li

**Affiliations:** 1 School of Information and Control Engineering, China University of Mining and Technology, Xuzhou, China; 2 School of Mechanical and Electrical Engineering, China University of Mining and Technology, Xuzhou, China; 3 Department of Pediatric, Yangpu District Shidong Hospital, Shanghai, China

**Keywords:** convolutional neural network, features fusion, multi-head attention, single channel electroencephalogram, sleep stage classification

## Abstract

**Introduction:**

Automated sleep stage classification is crucial for diagnosing sleep disorders and evaluating sleep quality. Single-channel EEG sleep staging using central electrodes has garnered significant interest due to its ability to detect a higher number of sleep spindles and its cost-effectiveness. Nevertheless, several challenges remain, such as the neglect of interdependencies among features, reduced model sensitivity to the dispersion across features, potential information leakage during the learning of transition rules, and class imbalance.

**Methods:**

To address these challenges, this study specifically designs an automatic sleep staging network based on single-channel EEG. The network incorporates a multi-branch feature extraction module, which captures negatively correlated frequency features that exhibit significant variations during sleep transitions and promotes synergistic complementarity. Furthermore, a dual-granularity residual attention mechanism is employed to perform adaptive fusion along both temporal and channel dimensions based on statistical measures such as the mean and standard deviation. The causal transition-aware network ensures temporal consistency while facilitating effective learning of transition rules. The focal loss function is utilized to counteract class imbalance.

**Results:**

Experiments on SleepEDF-20, SleepEDF-78 and SHHS datasets show that our method achieves the best performance with overall accuracy of 87.0%, 83.4% and 84.5%, respectively. For N1 stage classification which is more challenging, it reaches an accuracy of 57.5%, 52.5% and 49.3% on three datasets, respectively.

**Discussion:**

The experimental results demonstrate the effectiveness of the proposed approach and its certain advantages over existing methods.

## 1 Introduction

Sleep is crucial for human health, memory consolidation, and quality of life (Ramar et al., 2021). Sleep deprivation adversely impacts mental health and cognitive function in the short term. Prolonged sleep deficiency not only induces neuronal damage and impairs nervous system functionality, but also elevates the risk of cardiovascular and other diseases (Freeman et al., 2020; Luyster et al., 2012; Palagini et al., 2022). Given the pervasive global issue of sleep deficiency, there is a pressing need for effective sleep monitoring technologies to address this phenomenon, such as sleep stage classification (Grandner, 2020). Currently, the standard method for clinical sleep monitoring is the polysomnography (PSG), which synchronously collects key physiological signals such as electroencephalogram (EEG), electrooculogram (EOG), et al. through surface electrodes (Vaughn and Giallanza, 2008). According to the rules set by the American Academy of Sleep Medicine (AASM) (Berry et al., 2017), sleep stages are divided into five categories: wakefulness (W), rapid eye movement (REM), non-rapid eye movement 1 (N1), non-rapid eye movement 2 (N2), and non-rapid eye movement 3 (N3). Professional physicians need to classify each 30-s PSG signal into one of the five stages. Although this method is highly accurate, the process is cumbersome and time-consuming, imposing a heavy burden on professional physicians. Moreover, differences in physicians' diagnostic experience may cause inter-rater variability in sleep stage classification (Hirshkowitz, 2016). Therefore, current research efforts are increasingly directed toward the development of automated sleep stage classification methods.

Many researchers use traditional machine learning algorithms, such as support vector machine (SVM) (Rahman et al., 2018), k-nearest neighbors (KNNs), decision tree (Hassan and Bhuiyan, 2016), and random forest classifier (Rahman et al., 2018). For example, Madhuchhanda et al. (2026a) employs XGBoost to fuse traditional handcrafted descriptors with deep temporal-spatial representations, thereby improving model robustness and interpretability. However, traditional machine learning approaches have inherent limitations: feature extraction relies heavily on prior knowledge, the process is labor-intensive, and the effectiveness of the model largely depends on the quality of the selected features. In recent years, deep learning has been widely applied in automatic sleep staging due to its powerful feature representation and autonomous learning capabilities. Meanwhile, the combination of deep learning with single-channel EEG has attracted considerable attention because single-channel devices are low cost and suitable for home-based and wearable real-time monitoring. Most studies use a single channel containing central electrodes since sleep spindles predominantly appear in the central region (Andrillon et al., 2011), which provides a theoretical basis for channel selection.

Extracting multi-band features from mixed-mode EEG signals constitutes a critical prerequisite for meeting the discriminative criteria across different sleep stages. Convolutional neural networks (CNNs) have been widely adopted by researchers due to their capability in extracting time-invariant features from signals. Supratak et al. (2017) used two different sized convolution layers to extract invariant features from each raw single channel EEG signal. Zhou et al. (2022) adopted multiple convolution layers and multiple max average pooling layers to extract features at different scales from the EEG signals. Chambon et al. (2018) proposed a multivariate network architecture that uses linear spatial filters and convolutional layers to capture spectral features of physiological signals. Wei et al. (2023) decomposed the time-frequency images into three channel components (R, G, B), which were then fed into a dual-branch convolutional network, thereby opening up a new approach for processing EEG signals. Cong et al. (2025) employed convolutions with different dilation rates to extract temporal domain information, thereby forming features of multi-view EEG signals. Sleep is a dynamic process characterized by dynamic interactions among different EEG waves. Notably, δ waves exhibit the most pronounced negative correlations with other EEG frequencies, and these correlations vary systematically across sleep stage transitions, which provides valuable information for models to determine transition boundaries (Lin et al., 2020a). However, existing methods ignore the interrelationships among EEG components, which reduces the model's sensitivity to transition boundaries.

The fusion of multi-band features enhances the interdependencies among features, which facilitates accurate sleep stage classification by the model. The squeeze-and-excitation (SE) network (Hu et al., 2018) has attracted the attention of researchers due to its ability to enable information interaction between feature channels. Eldele et al. (2021) employed the SE network to establish dependencies between features from different branches of multi-scale convolutions, while introducing residual connections to form a hierarchical information structure. Zhou et al. (2024) introduced a residual SE network to enhance the features extracted by multiple convolutional layers and multiple max average pooling layers for feature fusion. Cao et al. (2026) also introduced a residual SE network to enhance feature dependency relationships, with the distinction that they performed information interaction simultaneously during feature extraction. Although these methods consider channel-level information, they overlook the temporal correlation among features. In addition, this pooling-based attention reduces the model's sensitivity to the dispersion of the distribution across channels, thereby degrading its accuracy.

The approximately 90-min sleep cycle consists of alternating periods of non-rapid eye movement (NREM) and REM sleep (Quan et al., 1997), during which the EEG transitions from high-amplitude slow waves to low-amplitude mixed frequencies. This evolutionary pattern demonstrates the critical role of temporal information in determining the accuracy of sleep staging. As a result, gated recurrent units (GRU) and long short-term memory (LSTM) networks have been widely used. Chen et al. (2020) proposed an attention mechanism-based CNN-LSTM fusion method that can automatically learn deep features from data with a high sampling rate, effectively improving classification performance. Madhuchhanda et al. (2026b) extracts classical features such as power spectral density (PSD) and combines them with a parallel architecture of LSTM and CNN. This design simultaneously captures the temporal dependencies and spatial representations of EEG signals, achieving good classification performance across most sleep stages. Kwon et al. (2021) employed an attention-enhanced bidirectional LSTM to capture temporal dependencies during sleep stage transitions and enhance the identification of critical sleep events through dynamic feature weighting. Cui et al. (2023) used convolutional layers to extract local features while using bidirectional gated recurrent units (BiGRU) to capture bidirectional contextual information, thereby modeling long-term dependencies and improving overall model performance. Zhao et al. (2022) proposed SleepContextNet, which combines CNN and RNN architectures to simultaneously capture both long-term and short-term temporal dependencies across sleep stages.

In recent years, the Transformer architecture has been introduced into sleep staging tasks due to its powerful global modeling capability, aiming to overcome the sequence length limitations of RNNs. For example, Sun et al. (2023) employs a Transformer-based approach for cross-modal fusion, learning joint representations from EEG and EOG signals for sleep classification. Zhang et al. (2024) first employed multi-scale CNN and adaptive channel calibration module to extract local features of EEG signals, followed by utilizing Transformer to capture global temporal contextual features, achieving excellent results. Sun et al. (2026) utilizes a Transformer to enhance the representation of both local and global features, thereby improving model performance. Ma et al. (2025) processed multi-channel PSG signal data to capture its complex spatiotemporal pattern characteristics, combining the Vision Transformer architecture with a supervised contrastive learning strategy for sleep staging classification. Although these methods exploit temporal structure to capture how features evolve over time, their feature embedding strategies for input time series inadvertently leak future information. This practice breaks temporal consistency, a critical factor that can adversely affect the model's generalization ability.

To address the class imbalance problem in sleep staging beyond model selection, the scarcity of N1 samples often leads to low precision for this stage in most methods. Hu et al. (2025) proposed a categorical cross-entropy and Wang et al. (2025) adopted weighted cross-entropy as loss functions. Despite certain improvements, these approaches require manually setting a weight for each class, which introduces many tuning parameters and requires careful adjustment across datasets with different class distributions.

In summary, the features extracted by existing methods lack interdependencies, and their feature fusion strategies fail to simultaneously consider both micro-level waveform characteristics and macro-level channel dependencies. These limitations impair the model's ability to discriminate transition boundaries, resulting in lower classification precision for minority classes such as N1. Regarding sleep transition rule learning, current approaches often leak future information, which violates temporal consistency and consequently compromises the model's generalization capability. For class imbalance issues, most methodologies require meticulous adjustment of weighting parameters tailored to specific datasets.

To address the above issues, an automatic sleep staging network specifically designed for single-channel EEG signals is proposed in this paper. The network consists of three modules: feature extraction, feature fusion, and temporal context awareness. The feature extraction module employs kernels of varying sizes to extract features at different frequencies. Each branch specifically targets distinct frequency band characteristics that exhibit significant negative correlation during sleep stage transitions, while the complementary focus across branches forms collaborative features. The feature fusion module implements a dual-granularity residual attention mechanism for adaptive fusion across both temporal and channel dimensions, simultaneously capturing microscopic waveform patterns and macroscopic channel dependencies. The temporal context awareness module incorporates a causal transition-aware network (CTN) to enhance temporal consistency and learn transition rules. Finally, focal loss is adopted as the loss function to accomplish the classification task.

The main contributions can be summarized as follows:

- We propose a multi-scale frequency anti-correlation convolutional network (MSFA-CN) to extract time-frequency features that exhibit significant negative correlation during sleep stage transitions. Meanwhile, features within the same branch are inter-correlated, while features across different branches complement each other, thereby enhancing the module's sensitivity to sleep transition patterns.
- We propose a dual-granularity residual attention (DRA) that focuses on micro-transient waveforms along the temporal dimension and macro-level channel dependencies along the channel dimension, thereby achieving adaptive feature fusion. Meanwhile, the introduction of residual connections effectively alleviates the vanishing gradient problem in deep models.
- The experimental results on the public datasets SleepEDF-20, SleepEDF-78, and SHHS demonstrate the superiority of the proposed method, with precision exceeding existing approaches for the challenging N1 stage. Additionally, ablation experiments qualitatively and quantitatively validate the effectiveness of each module.

## 2 Methodology

### 2.1 Model overview

In this section, we will present a detailed description of the model, which consists of three components, as illustrated in Figure 1. A three branch MSFA-CN is employed to extract time-frequency features from the input. A DRA is designed to allocate weights along time and channel dimensions for feature fusion, incorporating residual connections to integrate multi-scale information, which not only mitigates overfitting in deep network training but also enhances gradient flow. And a CTN applies causal convolution to the fused features to enforce temporal consistency, guiding weight allocation for each attention head, while multi head attention (MHA) captures multi-scale temporal dependencies to adaptively recalibrate the causal convolution weights. Finally, the output is fed into a classifier for automatic sleep stage classification.

**Figure 1:**
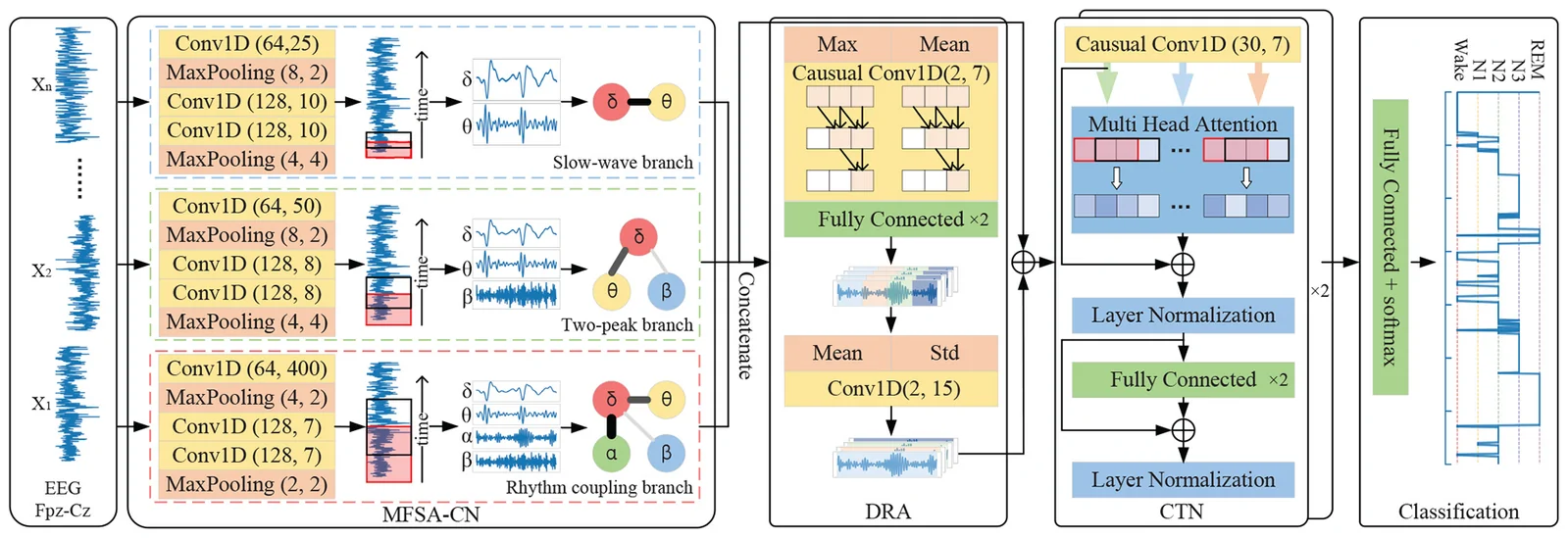
Overall framework of the proposed network.

### 2.2 Multi-scale frequency anti-correlation convolutional network(MSFA-CN)

Figure 2 shows the degree of negative correlation between different brainwaves across sleep stages. During transitions among wakefulness, REM, light sleep (LS), and deep sleep (DS), the negative correlation of with other brainwaves increases. Considering that different sleep stages are characterized by distinct frequency ranges, processing specific frequency bands is key to optimizing feature extraction. (Huang et al., 2019; Dai et al., 2017) Meanwhile, the degree of negative correlation among EEG components varies across sleep stage transitions, and capturing such negative correlations is beneficial for learning sleep transition rules. Therefore, we configured different convolutional kernel sizes in Figure 1 to select negatively correlated features from EEG signals across distinct frequency band perspectives, thereby specifically targeting the frequency bands associated with each sleep stage. The rationality of the kernel size selection can be illustrated through an example using a dataset with a sampling rate of 100 Hz (100 samples per second). The slow-wave branch's kernel size is 25: its 0.25 s time window can fully capture signal periods as low as 4 Hz. This range is below the lower bound of the important frequency band θ in sleep analysis and adjacent to the upper bound of the frequency band δ, which is helpful for capturing high-frequency activity features related to REM and state transitions. The two-peak branch's kernel size is 50: its 0.5 s time window can fully capture signal periods as low as 2 Hz, mainly covering the δ and α frequency bands. Consequently, this configuration is well-suited for distinguishing the W stage from the N1 and N2 stages. The rhythm coupling's kernel size is 400, and its 4 s time window can fully capture signal periods as low as 0.25 Hz (T = 1/F), matching the wave of δ. This extended window enables the integration of information across multiple frequency bands, thereby supplementing the global information. Finally, the features from different branches are concatenated, and two one-dimensional (1D) convolutions with kernel size 1 are employed to enforce the model to exploit negative correlation patterns between branch responses during the fusion process for distinguishing transitional phases, while simultaneously reducing dimensionality to minimize redundant information.

**Figure 2:**
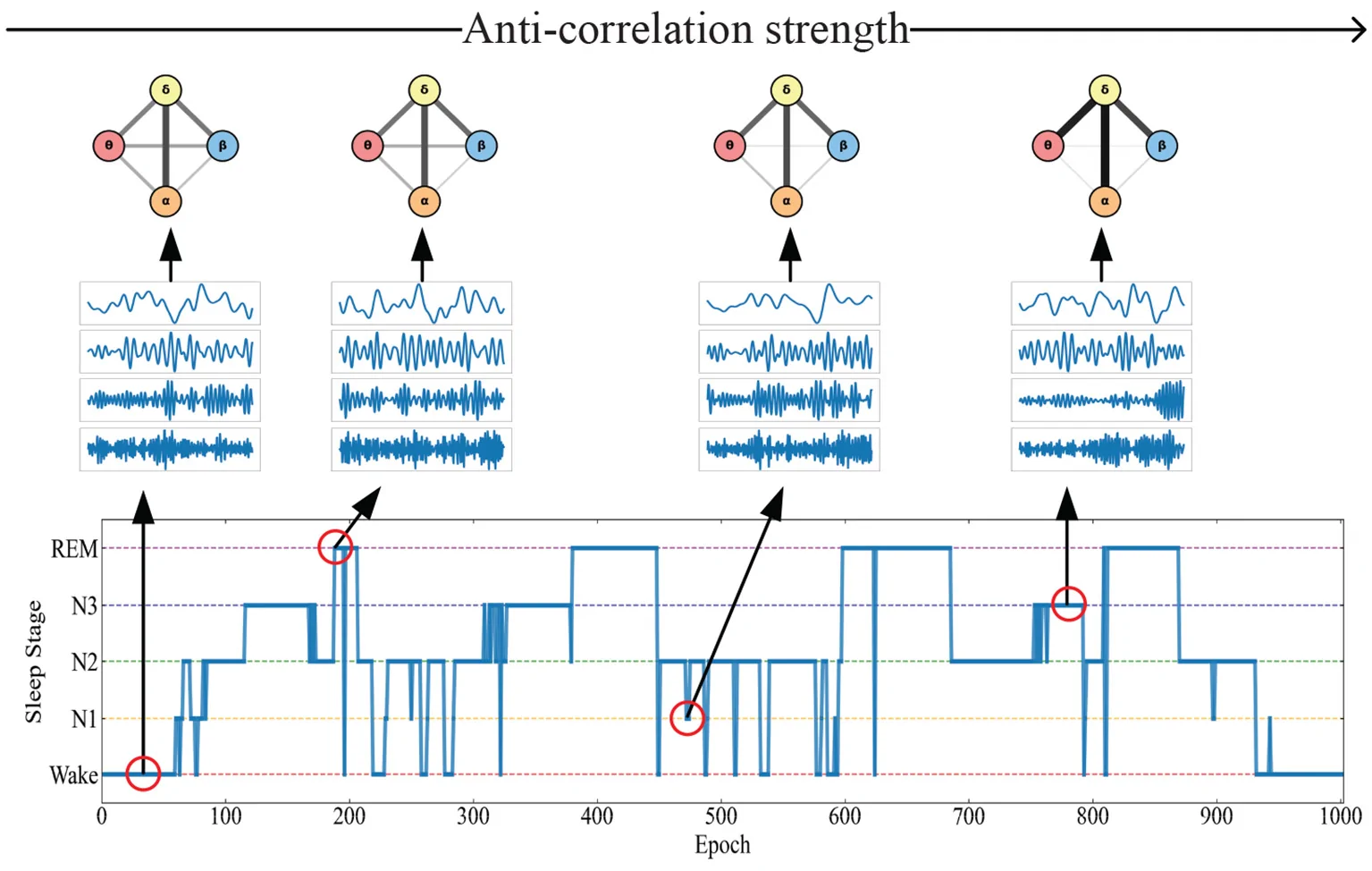
The strength of negative correlation of EEG components in each sleep stage.

Moreover, each convolutional branch consists of three convolutional layers and two max pooling layers, and uses the gaussian error linear unit (GELU) as the activation function to retain the negative amplitude features of the brain electrical signals. The notation Conv1D (64,50) in the figure represents a 1D convolutional layer with 64 filters, and a kernel size of 50, MaxPooling (8,2) represents a max pooling layer with a kernel size of 8 and a stride of 2. To prevent overfitting, we added batch normalization layers after each convolutional layer, and applied Dropout after the first max pooling in each branch and after the fusion of branch features.

### 2.3 Dual-granularity residual attention(DRA)

The DRA is one of the core components of this network architecture, specifically designed to integrate and enhance the multi-dimensional time-frequency features of the EEG signals in polysomnography. This is crucial for accurately differentiating between different sleep stages.

The DRA combines the temporal attention and channel attention mechanisms, aiming to focus separately on the dynamic time evolution patterns of EEG signals and the contribution of different channels, thereby modeling the complex dependencies between features and significantly improving the network's ability to represent sleep characteristics. Its overall structure is shown in Figure 3. Let MSFA-CN learn the feature mapping *I*∈*R*^*C*×*d*^, where *C* is the total channel of features and *d* is the feature length. First, for the characteristic of the dynamic changes of sleep EEG signals over time, such as the appearance of alpha waves, K-complex waves, and fluctuations in slow-wave activity, the time attention is used to extract the average and maximumvalues of the channel dimensions of the EEG signals, obtaining two feature maps $I_{avg}^{d}$ and $I_{max}^{d}$ hat characterize the global statistical quantities of the time dimensions. Then, along the channel dimension, these two feature mappings are connected, and a 1D causal convolution is used to capture the causal evolution trend of the extracted features along the time axis. This convolution operation ensures that the output depends only on the current and past time steps of the input, resulting in a feature map *I*^*d*^. The process can be expressed as follows:


$$\begin{array}{l} I^{d}=CausalConv1D\left(\left[I_{avg}^{d};I_{\max}^{d}\right]\right) \end{array}$$ (1)

where *CausalConv*1*D* represents a 1D causal convolution. Finally, to learn complex nonlinear interactions between channels, two fully connected (FC) layers are applied to obtain temporal weights *s*. Among them, the first layer is followed by a ReLU activation function for dimensionality reduction, and the second layer is followed by a smooth sigmoid activation function for increasing dimensionality and generating normalized temporal attention weights. The process can be expressed as follows:


$$\begin{array}{l} s=\sigma \left(W_{2}\left(\delta \left(W_{1}\left(I^{d}\right)\right)\right)\right) \end{array}$$ (2)

where σ, δ represents the sigmoid and ReLU activation functions respectively, and *W*_1_, *W*_2_ denote two FC layers. The original feature map *I* is scaled to the temporal attention feature map *F*∈*R*^*C*×*d*^ according to:


$$\begin{array}{l} F=I⊗s \end{array}$$ (3)

where ⊗ represents the dot product. The temporal attention feature map *F* is then fed into the channel attention. The channel attention calculates the average value and standard deviation for each channel of the features, resulting in two 1 × *C* feature maps $F_{avg}^{C}$, $F_{std}^{C}$. Then, these two features are mapped and connected along the channel dimension, and they are aggregated through a 1D convolution layer to reduce the number of channels to 1. Finally, the sigmoid activation function is applied to obtain the channel attention weights *c* as follows:


$$\begin{array}{l} c=\sigma \left(Conv1D\left[\left(F_{avg}^{C};F_{std}^{C}\right)\right]\right) \end{array}$$ (4)

where *Conv*1*D* represents the 1D convolution. Then, scale the *F* by *c* to obtain the feature image *O*∈*R*^*C*×*d*^:


$$\begin{array}{l} O=F⊗c \end{array}$$ (5)

At the end of DRA, a residual connection was introduced, adding the original input *I* to the features that were selectively enhanced by the DRA. The aim was to retain the multi-level features. Therefore, *X*∈*R*^*C*×*d*^, the fused feature of the DRA is defined as:


$$\begin{array}{l} X=I+O \end{array}$$ (6)

DRA employs causal convolution to model the temporal evolution trends of EEG signals and introduces a statistical combination of mean and standard deviation, thereby enhancing sensitivity to the dispersion degree of channel distributions. These design choices make DRA more sensitive than traditional pooling-based attention mechanisms, enabling superior capture of contextual relationships among EEG features.

**Figure 3:**
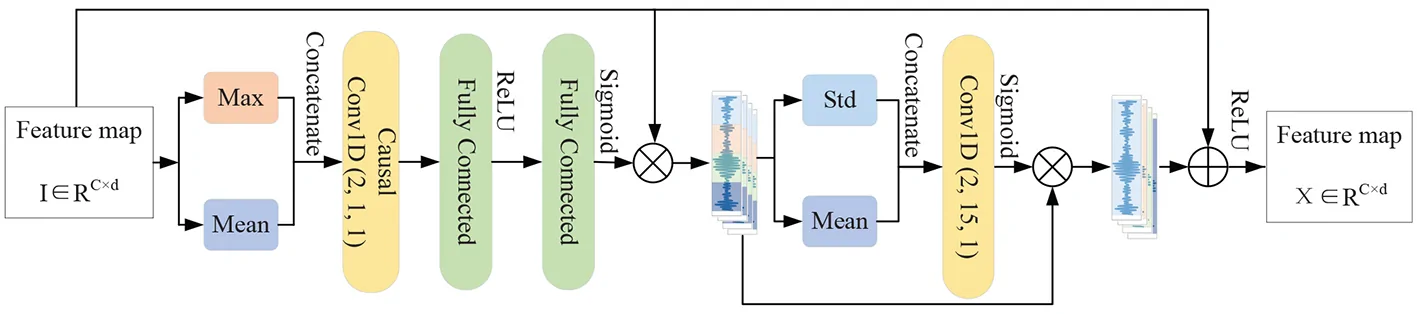
The strength of negative correlation of EEG components in each sleep stage.

### 2.4 Causal transition-aware network (CTN)

The core objective of CTN is to capture the temporal dependencies within the input features. In Figure 1, this layer consists of a MHA (Vaswani et al., 2017) module, two normalization layers (Ba et al., 2016), and two FC layers. To fully establish the relationship between the feature context, CTN stacks two identical above-mentioned structures to generate the final output features. Given that the attention mechanism is the core of CTN, we first explain the principle of causal MHA mechanism, and then detail the components of the CTN layer.

The MHA leverages an attention mechanism to perceive context within a 90-second window and capture latent variations across sleep stage transitions. Its core process is illustrated in Figure 4. First, causal convolution is applied to the input sequence to generate query, key, and value matrices, which are then split into multiple subspaces. Within each subspace, the importance of time steps is computed independently. Outputs from all subspaces are concatenated and linearly transformed to integrate contextual relationships among features. The key advantages of this structure are as follows. By generating query, key, value via causal convolution and dividing them into subspaces, it independently evaluates temporal importance, thereby enhancing focus on different positions and capturing temporal patterns. Moreover, multi-subspace projection improves feature representation flexibility and parallelism, significantly reducing training time compared to RNNs while effectively fusing local details with global temporal dependencies.

**Figure 4:**
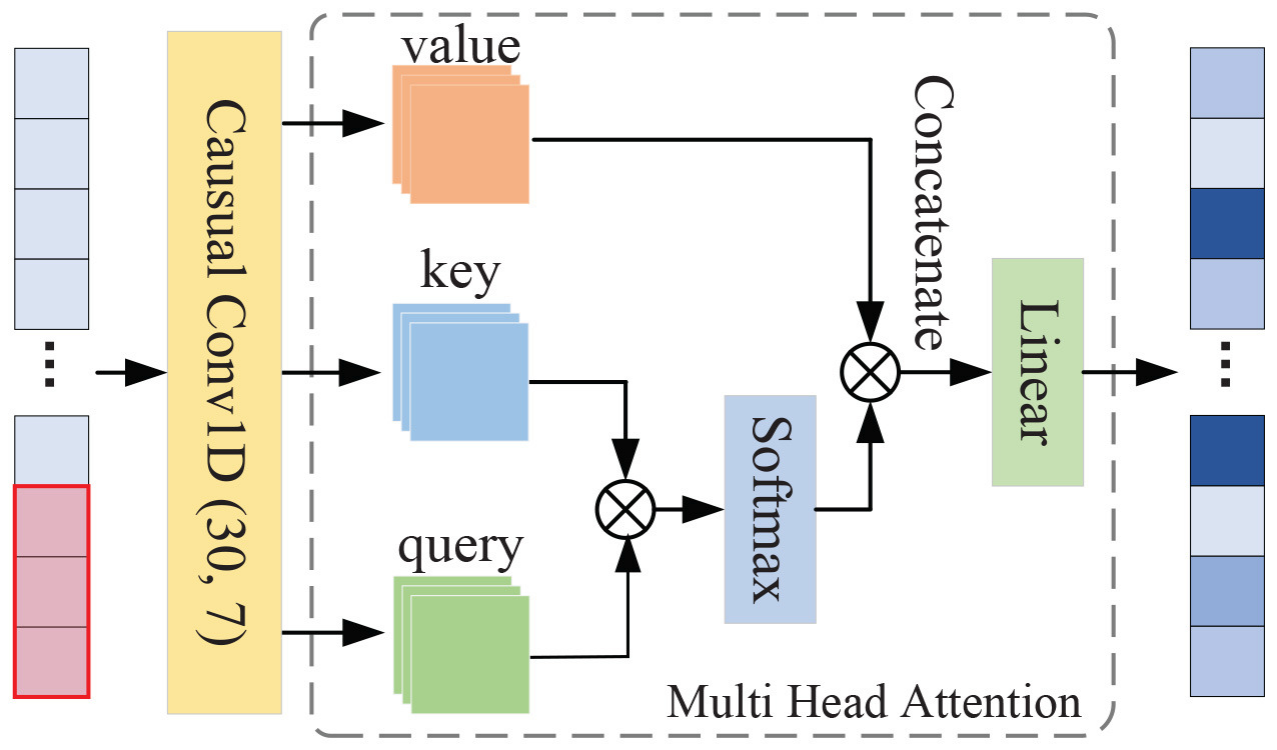
Schematic diagram of MHA perceiving temporal context.

The correlation weight score between the *i*-th position and the *j*-th position is calculated as follows:


$$\alpha_{ij}=\frac{\exp\left(s_{ij}\right)}{\sum_{k=1}^{d}\exp\left(s_{ij}\right)}$$ (7)

$$\begin{array}{l} s_{ij}=\varphi \left(x_{i}\right)\varphi {\left(x_{j}\right)}^{T} \end{array}$$ (8)

where *s*_*ij*_ represents the correlation between them, and φ(·) is a causal convolution. Therefore, the weighted sum *a*_*i*_ of each context attention is calculated as:


$$\begin{array}{l} a_{i}=\overset{d}{\underset{j=1}{\sum }}\alpha_{ij}\varphi \left(x_{j}\right) \end{array}$$ (9)

The wight of the context attention layer can be represented by $A=\left(a_{0},...,a_{d}\right)\in R^{C\times d}$. The input of the causal MHA is the fused feature maps $X=\left(x_{0},...,x_{C}\right)\in R^{C\times d}$ of DRA, where *C* is the total number of features and *d* is the length of *X*_*i*_(1 ≤ *i* ≤ *C*). After undergoing spatial transformation through three independent causal convolutions with kernel size 7 and stride 1, the query, key, and value are obtained as inputs. The output of this causal convolution depends solely on the current and previous time steps to generate outputs, strictly preventing information leakage. As a result, the generalization ability of the model is enhanced. Through these three matrices, the attention in the following formula can be calculated according to (Vaswani et al., 2017):


$$\begin{array}{l} ATT\left(q,k,v\right)=Soft\max\left(\frac{qk^{T}}{\sqrt{d}}\right)v \end{array}$$ (10)

where *q*, *k*, *v* represent the query matrix, the key matrix, and the value matrix, respectively. The matrices of query, key, and value are first divided into multiple subspaces based on the number of heads of the MHA layer. Each subspace can focus on different parts of the input sequence and capture different patterns or features. This enables the network to better establish models with long-term dependencies and capture more complex relationships between different parts of the input. In each subspace, the context scores *A*^*h*^∈*R*^*C*×(*d*×*H*)^, where *H* is the head count of MHA, can be expressed by the following formula:


$$\begin{array}{l} A^{h}=ATT\left(q^{h},k^{h},v^{h}\right) \end{array}$$ (11)

where *q*^*h*^, *k*^*h*^, *v*^*h*^ represent the query, key, and value of the *h*-th(1 ≤ *h* ≤ *H*) subspace, respectively.

Finally, by concatenating the context scores of the subspaces, the final context aware can be obtained, which can be expressed as follows:


$$\begin{array}{l} MHA\left(q,k,v\right)=Concatenate\left(A^{1},...,A^{H}\right) \end{array}$$ (12)

Then, residual connections are employed to form multi-layer features, preventing gradient explosion and enhancing feature representability. Layer normalization is then applied to the sum to accelerate model training. This operation can be expressed as follows:


$$\begin{array}{l} Y\left(x\right)=x+\phi \left(x\right) \end{array}$$ (13)

where *Y*(*x*) represents the result of layer normalization, ϕ(·) refers to the MHA in Figure 1, or two FC layers, and *x* is the input of layer normalization. The use of residual connections can help the model utilize features from lower layers, prevent gradient explosion, and enhance the feature expression ability. Moreover, the layer normalization operation helps to accelerate the model training.

The context features, after residual connection and layer normalization, are fed into a feed-forward network. This network consists of two fully connected layers, in Figure 1. A ReLU activation function is used between the two fully connected layers to enhance the feature expression ability of the model, address the limitations caused by the previous linear operations on feature expression, and consider the potential representation of features. The process can be expressed as follows:


$$\begin{array}{l} M=W_{4}\left(\delta \left(W_{3}\left(x\right)\right)\right) \end{array}$$ (14)

where *M* is the output of the feedforward neural network, while *W*_3_ and *W*_4_ represent two fully connected layers, and δ represents the ReLU activation function.

### 2.5 Focal loss function

To address the issue of class imbalance in EEG data, we introduce focal loss (Lin et al., 2020b) as the loss function. This loss function was initially used in the image domain to solve the extremely unbalanced positive and negative samples and the problem of difficult classification sample learning in one-stage object detection. Focal loss balances the cross-entropy loss function and adds a regulating factor to reduce the weight of easy-to-classify samples, focusing on the training of difficult-to-classify samples. Its formula is as follows:


$$\begin{array}{l} FL\left(p_{t}\right)=-\alpha {\left(1-p_{t}\right)}^{\gamma }\log\left(p_{t}\right) \end{array}$$ (15)

where the hyperparameters α and γ can adjust the behavior of the model. Parameter α is used to regulate the impact of class imbalance, while the focusing parameter γ controls the weight of the model for easily classified samples. The larger the value of γ, the lower the model's attention to such samples. *p*_*t*_ represents the predicted probability of the true class by the softmax layer, and its value reflects the classification difficulty of the sample: the smaller the *p*_*t*_, the more difficult it is to distinguish the sample, and the higher the corresponding loss weight. This mechanism aims to enhance the model's attention to difficult-to-distinguish samples in the minority class and effectively alleviate the problem of class imbalance. Different from the traditional weighted cross-entropy loss, which mainly focuses on the class level, focal loss focuses on the sample level, thus eliminating the dependence of model performance on the distribution differences of sleep stages.

## 3 Experiment configuration

### 3.1 Experimental setting

The proposed network chooses *h* = 5 for the number of heads of MHA. The parameters of the focal loss are chosen α = 2 and γ = 2 for the optimization of the loss function. We applied a batch size of 128 and a random seed of 123. And the Adam optimizer with the learning rate starts with 1e-3 and then reduces to 1e-4 after ten epochs. And the weight decay of Adam is set to 1e-3. All the convolutional layers are initialized using a Gaussian distribution with a mean of 0 and a variance of 0.02. Finally, the network is trained on the 24 GB of NVIDIA RTX 3090 GPU.

### 3.2 Dataset

Three public datasets were used in this experiment: SleepEDF-20, SleepEDF-78, and SHHS. SleepEDF-20 and SleepEDF-78 are from PhysioBank (Goldberger et al., 2000). We used the sleep cassette data, which investigates age effects on sleep in healthy participants aged 25–101 years. In the polysomnography recordings, each 30-s epoch was labeled as one of: Stage 0, Stage 1, Stage 2, Stage 3, Stage 4, REM, sleep latency, or “?” (unscored). For this study, these annotations were converted to AASM standards: Stages 3 and 4 were merged into N3, while Stages 0, 1, and 2 were relabeled as W, N1, and N2, respectively. The labels “sleep latency” and “?” were excluded as they do not belong to the five sleep stages. In addition, the dataset only includes wake periods from 30 minutes before sleep onset to 30 minutes after sleep end to enhance focus on sleep stages.

SHHS (Quan et al., 1997; Zhang et al., 2018) is a multicenter longitudinal study investigating the effects of sleep-disordered breathing on cardiovascular disease. Subjects had various conditions, including pulmonary, cardiovascular, and coronary artery diseases. To minimize the influence of these diseases and of moderate to severe sleep-disordered breathing in clinical settings, we selected subjects with regular sleep patterns (for example, apnea-hypopnea index or AHI < 5). SHHS was originally annotated according to R&K guidelines, and we converted these annotations to AASM using the same approach as for SleepEDF.

Table 1 shows the detailed information of the three datasets, including sampling rate, channels used, and number of subjects.

**Table 1:** Number of 90s epochs for each sleep stage from experimental datasets.

Dataset	Subjects	EEG channel	Sampling rate	W	N1	N2	N3	REM	Total
SleepEDF-20	20	Fpz-Cz	100Hz	8207 19.4%	2804 6.6%	17799 42.1%	5703 13.5%	7717 18.3%	42230
SleepEDF-78	78	Fpz-Cz	100Hz	65646 33.6%	21521 11.0%	69132 35.4%	13039 6.7%	25835 13.2%	195173
SHHS	329	C4-A1	125Hz	45811 14.1%	10291 3.2%	142035 43.8%	60140 18.6%	65919 20.3%	324196

### 3.3 Data preprocessing

Most of the work uses a single 30-s window as the input, and then predicts a label. Although this method is simple, it ignores the correlations and dependencies existing between the surrounding windows. Meanwhile, in practical applications, when professionals cannot fully determine the sleep stage of a single 30-s window, they often combine the adjacent two windows for the final judgment. In this study, we combine the input of a single 30-second window from each subject with adjacent windows, expanding it to form a contextual input. The preprocessing process is shown in Figure 5. Each context input consists of three adjacent windows, namely the first 30-s window *x*_*n*−1_, the middle 30-s window *x*_*n*_, and the last 30-s window *x*_*n*+1_, forming context input *X*_*n*_, and the label of the middle 30-s window *y*_*n*_ is used as the label of the context input.

**Figure 5:**
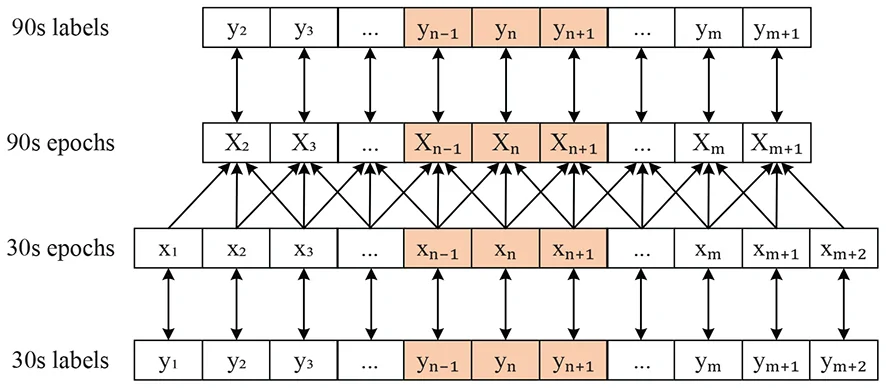
Schematic diagram of the 90s epochs and labels.

Table 1 shows the number of context inputs for each sleep stage after data preprocessing in the SleepEDF-20, SleepEDF-78, and SHHS datasets. The five stages exhibit an uneven distribution. For all datasets, the W and N2 stages account for more than 50% of all windows. In contrast, the N1 and N3 stages account for the smallest proportion, approximately 20% of all windows in total.

### 3.4 Evaluation indicators

This experiment uses four evaluation metrics to measure the classification effectiveness of the network, namely accuracy (ACC), macro F1 score (MF1), and kappa (Kohen, 1960). MF1 is commonly used indicators for evaluating the performance of models on imbalanced datasets (Shen et al., 2023). Based on the confusion matrix, we can obtain the true positives (*TP*_*i*_), false positives (*FP*_*i*_), true negatives (*TN*_*i*_), and false negatives (*FN*_*i*_) for the *i*-th class. From these, the accuracy (*PR*_*i*_), recall rate (*RE*_*i*_), and specificity (*SP*_*i*_) can be calculated. The formulas are as follows:


$$\begin{array}{l} PR_{i}=\frac{TP_{i}}{TP_{i}+FP_{i}} \end{array}$$ (16)

$$\begin{array}{l} RE_{i}=\frac{TP_{i}}{TP_{i}+FN_{i}} \end{array}$$ (17)

$$\begin{array}{l} SP_{i}=\frac{TN_{i}}{TN_{i}+FP_{i}} \end{array}$$ (18)

Then the overall accuracy ACC and MF1 are defined as follows:


$$ACC=\frac{\sum_{i=1}^{k}TP_{i}}{M}$$ (19)

$$\begin{array}{l} MF1=\frac{1}{k}\overset{k}{\underset{i=1}{\sum }}\frac{2\times PR_{i}\times RE_{i}}{PR_{i}+RE_{i}} \end{array}$$ (20)

where *M* represents the total number of samples, and *k* is the number of categories or sleep stages.

## 4 Results and discussion

### 4.1 Sleep stage classification results

To facilitate a more intuitive observation of the model's performance in each category on the datasets, we provide a detailed description of the classification results through the confusion matrix, as shown in Figure 6. It can be seen that the N1 samples are prone to be confused with N2, REM, and W stages. This is due to the following reasons: N1 is the transitional stage from wakefulness to sleep, with a short process and being easily disturbed by external factors, resulting in a smaller number of samples and the model being unable to effectively learn the relevant features. Secondly, when the subject enters the N1 stage, the brain shifts from mainly generating high-frequency, low-amplitude α rhythm and β rhythm to low-frequency, high-amplitude θ rhythm. This process usually involves significant changes and is susceptible to external noise interference, making it difficult to distinguish. Although CTN mitigates isolated N1 misclassifications through context awareness by leveraging temporal consistency across contextual windows to constrain predictions, overlapping EEG features between N1 and REM stages, combined with the lack of EOG information in single-channel EEG to identify rapid eye movement characteristics of REM, constitute an inherent limitation of unimodal approaches.

**Figure 6:**
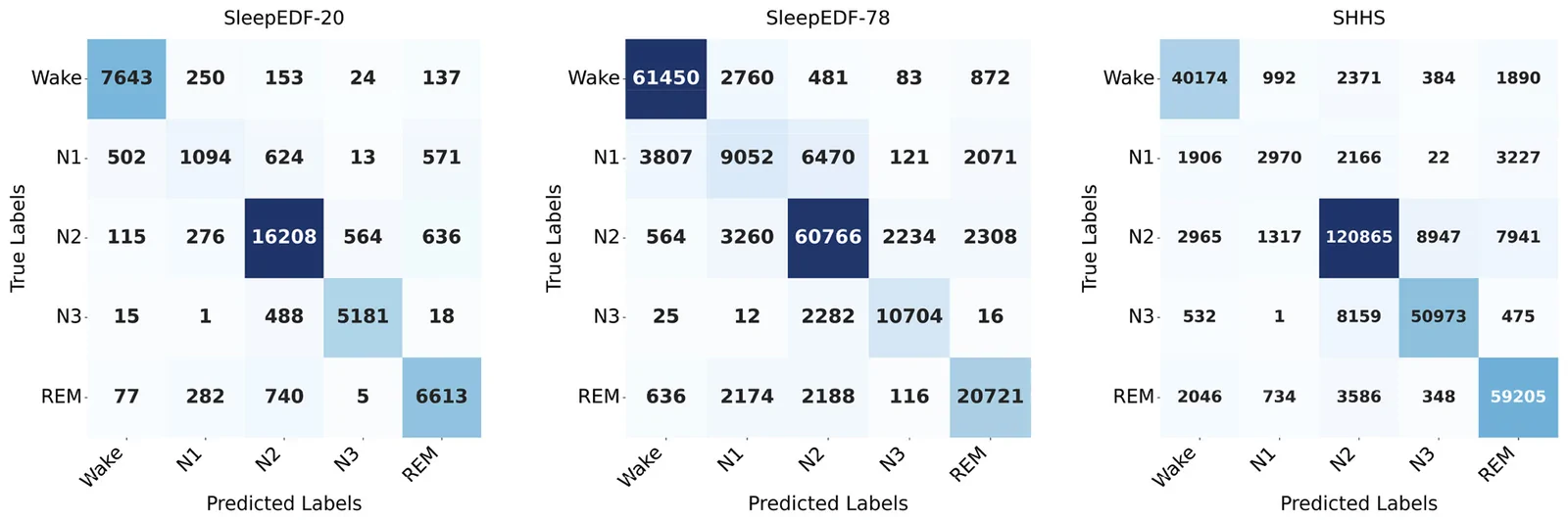
Confusion matrix heatmap of the model on different datasets.

### 4.2 Performance comparison

In Table 2, we compared the proposed model with some existing works using the same dataset in terms of accuracy, macro F1, and kappa. We also conduct one-sample t-tests between the results of our model under 20-fold cross-validation and the published results to assess whether the improvements are statistically significant, as shown in Table 3. On SleepEDF-20, our model outperforms most models in Acc, MF1, and kappa, and remains competitive with several networks. In particular, it achieves Acc and kappa comparable to those of the multimodal-input network FlexibleSleepNet (Ren et al., 2025). Moreover, it attains higher classification accuracy in the N1 stage than other deep learning methods. This confirms that our model can better extract useful features from contextual inputs. Moreover, its classification accuracy for the N1 stage is also better than that of other deep learning methods. This confirms that, for contextual input, our model can better extract useful features. As a result, it improves the accuracy of the N1 stage, which is difficult for other models to classify. Among the single-channel input models, for the REM stage that requires electrooculogram signal input for distinction, our model also achieves a relatively high accuracy rate relying solely on single-channel EEG signals. On the SleepEDF-78 dataset, our model remains competitive. It also achieves the best classification accuracy in the N1 stage, which further demonstrates that our model can extract effective features from contextual inputs. For the larger SHHS dataset, our model also achieved excellent performance. Although the channel used in the SHHS dataset differs from that in SleepEDF, both datasets include central electrodes. The central region is the primary area where sleep spindles occur. This, to some extent, mitigates the impact of channel differences. The comparison results show that the framework proposed in this paper has achieved good performance on the SleepEDF-20, SleepEDF-78, and SHHS datasets.

**Table 2:** Comparison of the performance of the network with that of the state-of-the-art methods on the datasets SleepEDF-20, SleepEDF-78 and SHHS.

Dataset	Method	Acc	MF1	kappa	W	N1	N2	N3	REM
SleepEDF-20	Mourtazaev et al. (1995)	83.3^***^	77.3^***^	0.76^***^	-	-	-	-	-
	Supratak et al. (2017)	81.9^***^	76.6^***^	0.76^***^	86.7	45.5	85.1	83.3	82.6
	Mousavi et al. (2019)	81.5^***^	76.6^***^	0.75^***^	89.4	44.4	84.7	84.6	79.6
	Eldele et al. 2021)	84.4^***^	78.1^***^	0.79^***^	86.7	42.6	88.8	90.2	79.0
	Zhao et al. (2022)	84.8^***^	79.8^***^	0.79^***^	91.2	46.9	88.0	90.5	82.5
	Fu et al. (2023)	86.6^**^	80.4^**^	0.82	90.5	46.9	89.2	90.1	85.4
	Zaman et al. (2024)	86.3^***^	80.9	0.81^***^	89.5	51.0	90.2	91.4	79.4
	Huang et al. (2025)	85.4^***^	80.8	0.80^***^	85.3	55.6	91.3	90.1	81.8
	Ren et al. (2025)	86.9	81.9^***^	0.82	92.8	57.2	89.8	85.0	87.2
	Cong et al. (2025)	83.1^***^	75.0^***^	0.79^***^	87.9	33.5	87.5	85.8	80.3
	Ours	87.0	80.7	0.82	91.5	57.5	89.0	89.5	82.9
SleepEDF-78	Mourtazaev et al. (1995)	80.6^**^	76.7	0.73^**^	-	-	-	-	-
	Supratak et al. (2017)	77.8^**^	71.8^**^	0.70^***^	90.9	45.0	79.2	72.7	71.1
	Mousavi et al. (2019)	74.2^***^	69.6 ^***^	0.66^***^	89.9	42.1	75.2	70.4	70.6
	Eldele et al. (2021)	81.3^**^	75.1^**^	0.74^**^	92.0	42.0	85.0	82.1	74.2
	Zhao et al. (2022)	82.7	77.2	0.76	93.0	50.0	84.3	80.2	78.6
	Fu et al. (2023)	82.2^*^	73.6^**^	0.75^*^	88.5	48.4	81.9	78.6	80.0
	Huang et al. (2025)	83.4	76.2^*^	0.77	92.5	41.1	87.2	83.4	76.7
	Cong et al. (2025)	82.3^*^	74.7^**^	0.75^*^	90.9	39.7	83.2	76.6	73.5
	Ours	83.4	77.4	0.77	92.4	52.5	84.2	80.7	79.7
SHHS	Supratak et al. (2017)	81.0^**^	73.9^*^	0.73^**^	85.4	40.5	82.5	79.3	81.9
	Eldele et al. (2021)	84.2	75.3	0.78	86.7	33.2	87.1	87.1	82.1
	Zhao et al. (2022)	78.8^**^	72.7^**^	0.71^***^	88.8	51.4	87.0	86.6	89.0
	Huang et al. (2025)	86.2^*^	77.6^*^	0.81^**^	88.0	37.1	89.3	90.7	83.0
	Cong et al. (2025)	81.4^**^	71.2^**^	0.74^**^	82.2	25.7	83.9	83.3	81.1
	Ours	84.5	75.6	0.78	85.5	49.3	88.3	82.9	81.1

^***^p < 0.001; ^**^p < 0.01; ^*^p < 0.05.

**Table 3:** *P*-values of one-sample *t*-tests comparing the network's performance with state-of-the-art methods on the SleepEDF-20, SleepEDF-78, and SHHS datasets.

Dataset	Method	Acc	MF1	kappa
SleepEDF-20	Mourtazaev et al. (1995)	3.304e-07^***^	1.541e-06^***^	6.061e-08^***^
	Supratak et al. (2017)	8.831e-08^***^	7.498e-07^***^	6.061e-08^***^
	Mousavi et al. (2019)	6.484e-08^***^	7.498e-07^***^	3.247e-08^***^
	Eldele et al. (2021)	1.433e-06^***^	4.283e-06^***^	1.021e-06^***^
	Zhao et al. (2022)	2.891e-06^***^	1.993e-04^***^	1.021e-06^***^
	Fu et al. (2023)	6.982e-03^**^	5.241e-03^**^	2.541e-01
	Zaman et al. (2024)	4.535e-04^***^	4.993e-01	1.008e-04^***^
	Huang et al. (2025)	1.123e-05^***^	6.36e-01	5.439e-06^***^
	Ren et al. (2025)	7.314e-01	1.86e-04^***^	2.541e-01
	Cong et al. (2025)	2.659e-07^***^	2.086e-07^***^	1.021e-06^***^
SleepEDF-78	Mourtazaev et al. (1995)	4.445e-03^**^	1.354e-01	2.879e-03^**^
	Supratak et al. (2017)	1.009e-03^**^	1.526e-03^**^	8.733e-04^***^
	Mousavi et al. (2019)	2.494e-07^***^	1.668e-06 ^***^	1.704e-07^***^
	Eldele et al. (2021)	8.429e-03^**^	9.875e-03^**^	5.423e-03^**^
	Zhao et al. (2022)	1.237e-01	9.489e-01	7.755e-02
	Fu et al. (2023)	3.13e-02^*^	3.414e-03^**^	1.371e-02^*^
	Huang et al. (2025)	2.604e-01	4.086e-02^*^	2.323e-01
	Cong et al. (2025)	3.878e-02^*^	7.014e-03^**^	1.371e-02^*^
SHHS	Supratak et al. (2017)	2.63e-03^**^	1.316e-02^*^	1.533e-03^**^
	Eldele et al. (2021)	1.105e-01	1.207e-01	1.287e-01
	Zhao et al. (2022)	1.042e-03^**^	5.216e-03^**^	8.291e-04
	Huang et al. (2025)	1.65e-02^*^	1.834e-02^*^	7.758e-03^**^
	Cong et al. (2025)	3.297e-03^**^	2.431e-03^**^	2.279e-03^**^

^***^p < 0.001; ^**^p < 0.01; ^*^p < 0.05.

### 4.3 Ablation experiment

To quantitatively analyze the effectiveness of the MSFA-CN, the DRA, and the CTN in the network, an ablation study was conducted on the SleepEDF-20 dataset, as shown in Figure 7. This paper investigated the performance of four network variables on the SleepEDF-20 dataset, namely: Complete Network; Transform A: MSFA-CN; Transform B: MSFA-CN + CTN; Transform C: MSFA-CN + DRA.

**Figure 7:**
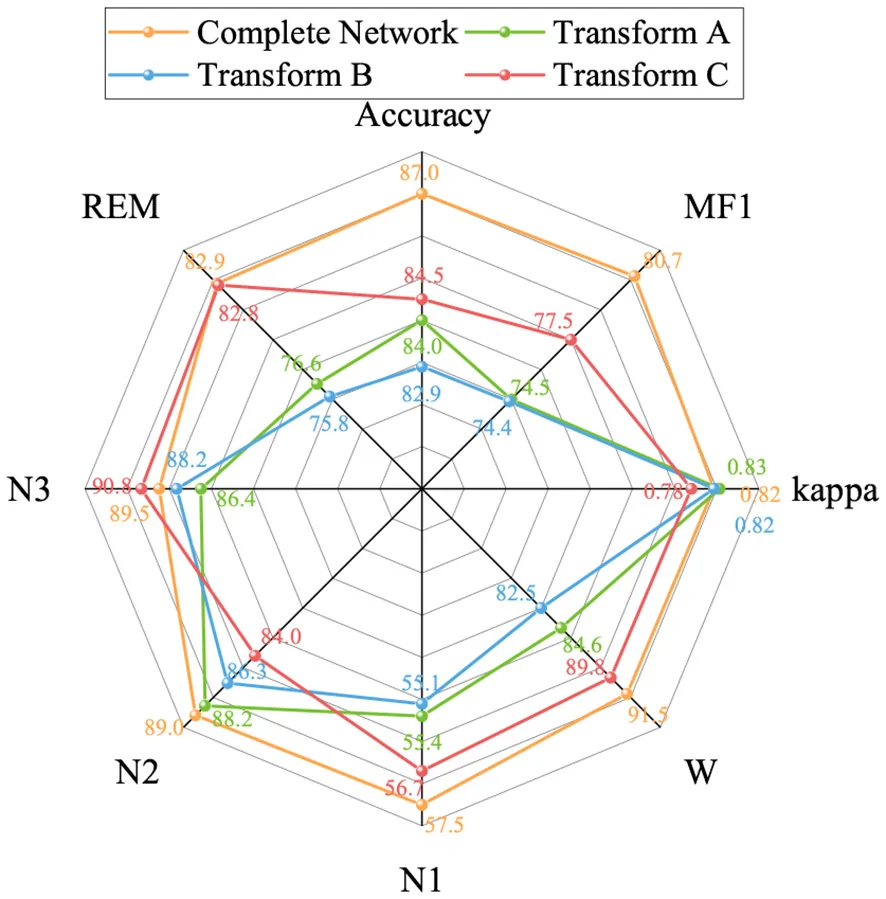
Performance comparison of ablation experiments.

Based on the above experimental results, we can draw the following conclusions. Firstly, DRA can improve the classification performance, which proves the necessity of fusing multi-level features. This point is further demonstrated by comparing Transform A and Transform C. Secondly, by comparing Transform C and the complete network, we conclude that capturing time dependence using CTN is important for sleep stage classification. Moreover, DRA is more important than CTN because the Transform C outperforms the Transform B. Thirdly, the MF1 and accuracy of the complete network are significantly better than those of the other three variants, indicating that all the constituent modules of the proposed overall network are indispensable. In terms of the classification performance of each sleep stage, the complete model is significantly better than the other three variants, and the classification performance of the N3 stage is comparable to that of the best variant.

Meanwhile, to qualitatively analyze the effectiveness of various networks, we use t-SNE (Hinton and Roweis, 2003) technology to map these features onto a two-dimensional plane. It is one of the most effective methods for dimensionality reduction and visualization of high-dimensional data. In this study, 20-fold cross-validation was conducted on SleepEDF-20 for 20 subjects. Therefore, in the experiment, one subject was randomly selected as the validation set and the remaining 19 subjects as the training set for each cross-validation. Figure 8 shows the feature visualization results of the first five-folds, with the subject numbers of the test set marked. Each fold result includes the original features and the features processed by four model variables: Transform A, Transform B, Transform C and the complete model. The number of points on each subgraph represents the number of input windows of the subjects, and different colors represent different sleep stages. By comparing Figures 8b, c, it can be seen that the feature distribution of Transform B is more scattered than that of Transform A, reflecting the inferior performance of Transform B compared to Transform A. Furthermore, compared with Figures 8b, d better represents the differences between samples because DRA further enhances feature expression. Compared with Figures 8d, e shows clearer boundaries between different sleep stages and reduced distances between samples of the same sleep stage. This is because the CTN captures contextual relationships between features, thereby classifying samples more effectively. It is worth noting that although t-SNE visualization can qualitatively assess the classification performance of a model based on the degree of clustering among features, it also has the limitation of being sensitive to hyperparameters.

**Figure 8:**
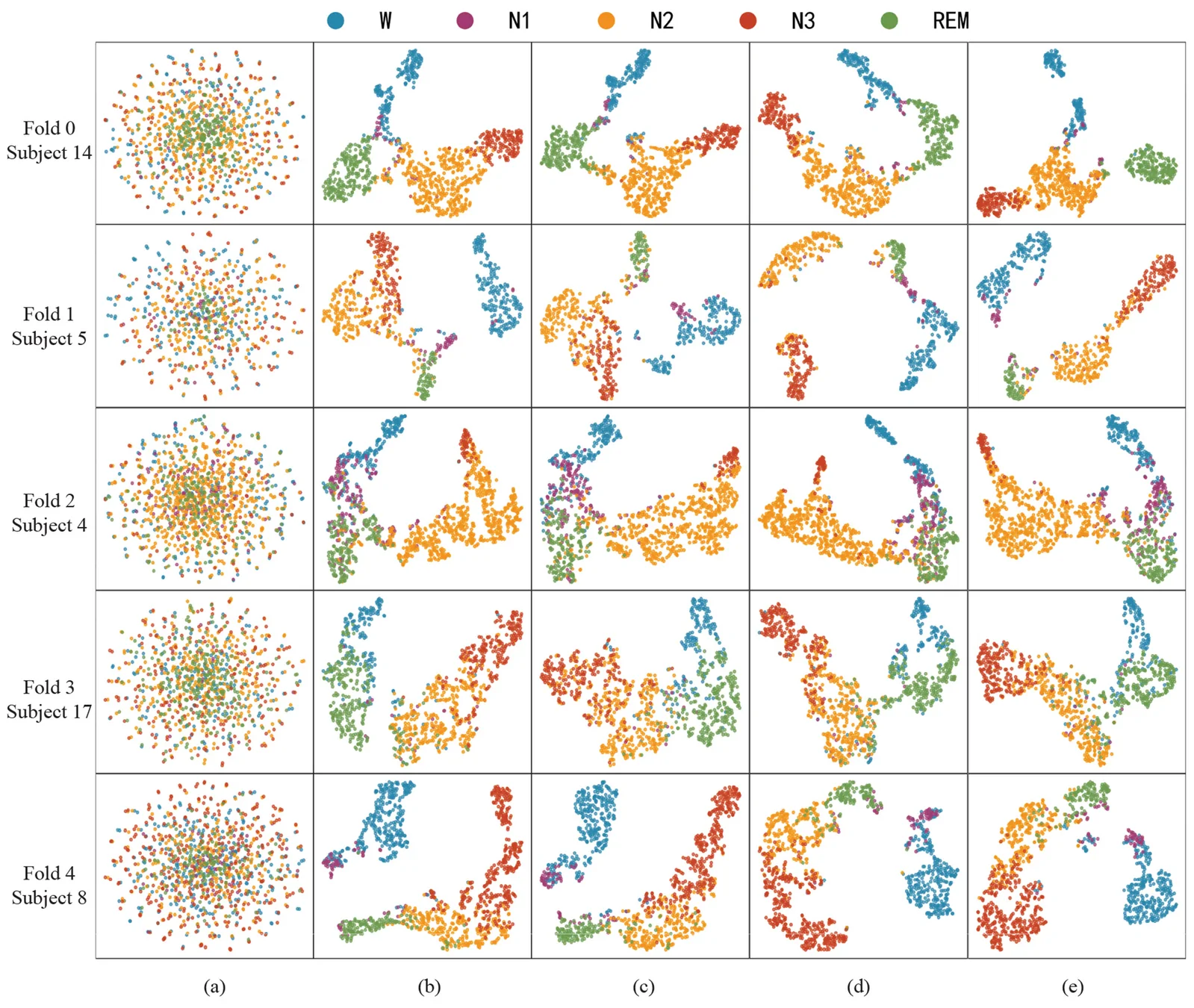
t-SNE feature visualization result graph, **(a)** original features, **(b)** Transform A, **(c)** Transform B, **(d)** Transform C, **(e)** complete network.

### 4.4 Analysis of prediction results

Figure 9 illustrates the actual and predicted sleep stage transitions for Subject 14 from SleepEDF-20. Subject 14 has a total of 1,002 epochs, with 71 misclassifications, achieving an accuracy of 92.9%. Errors are marked with red crosses in the figures. From Figure 9, it can be observed that classification errors predominantly occur during abrupt transitions between sleep stages, such as from W to N1 or N2, or during brief awakenings within sleep. Among all stages, N1 exhibits the highest error rate, with most misclassifications as N2, followed by W due to its large sample size. This indirectly supports the confusion matrix findings: W, N2, N3, and REM are well-classified, whereas N1 performs the worst. Moreover, our model effectively learns sleep transition rules, it reproduces patterns around frequent, abrupt N2 to N3 transitions, though this occasionally introduces misclassifications. Nevertheless, the model demonstrates strong overall performance in sleep staging.

**Figure 9:**
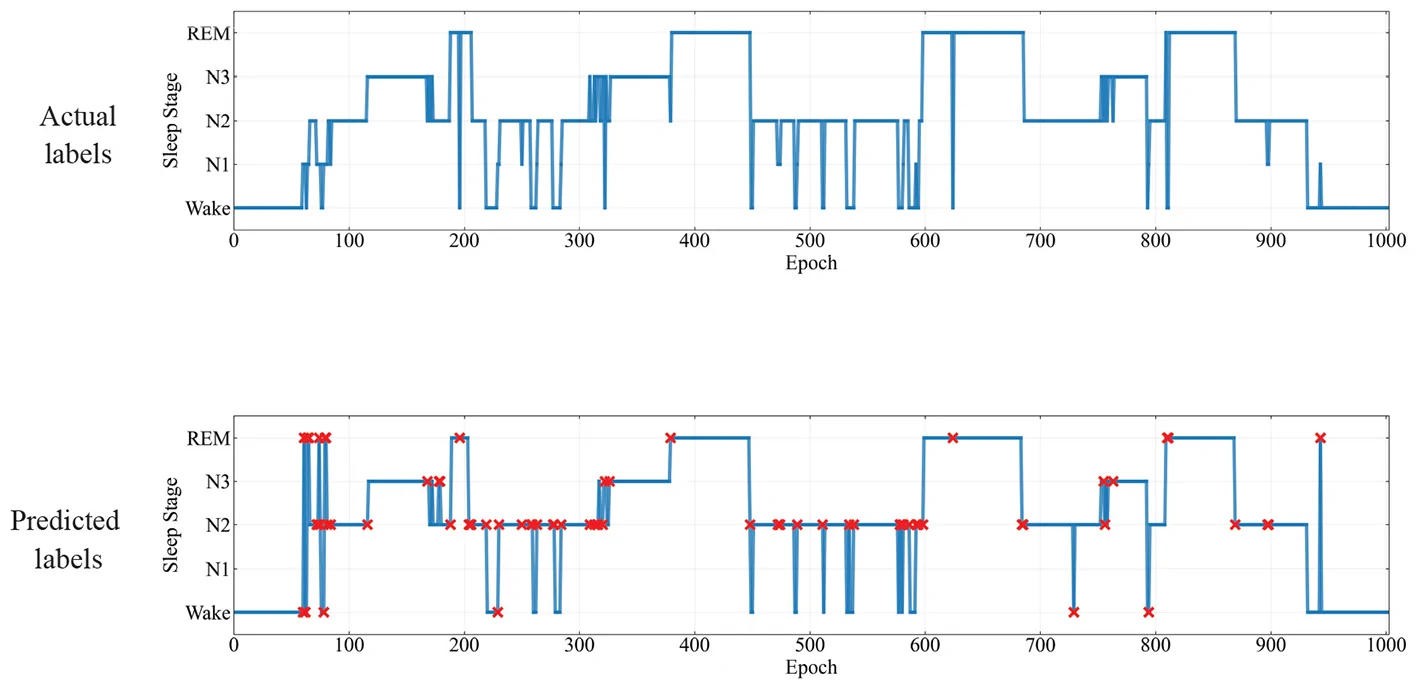
On the SleepEDF-20, a comparison chart of the predicted labels and actual labels on the validation set of random subject 14.

### 4.5 MSFA-CN frequency response analysis

To verify the theoretical feasibility of the MSFA-CN architecture, we visualized the response amplitudes of its three parallel branches to different EEG frequency bands. In Figure 10, the first five rows randomly present results from five-folds of the SleepEDF-20 dataset, with the frequency responses averaged across these folds to show the overall pattern. These branches exhibit clear differences and complementarity in their spectral response characteristics.

**Figure 10:**
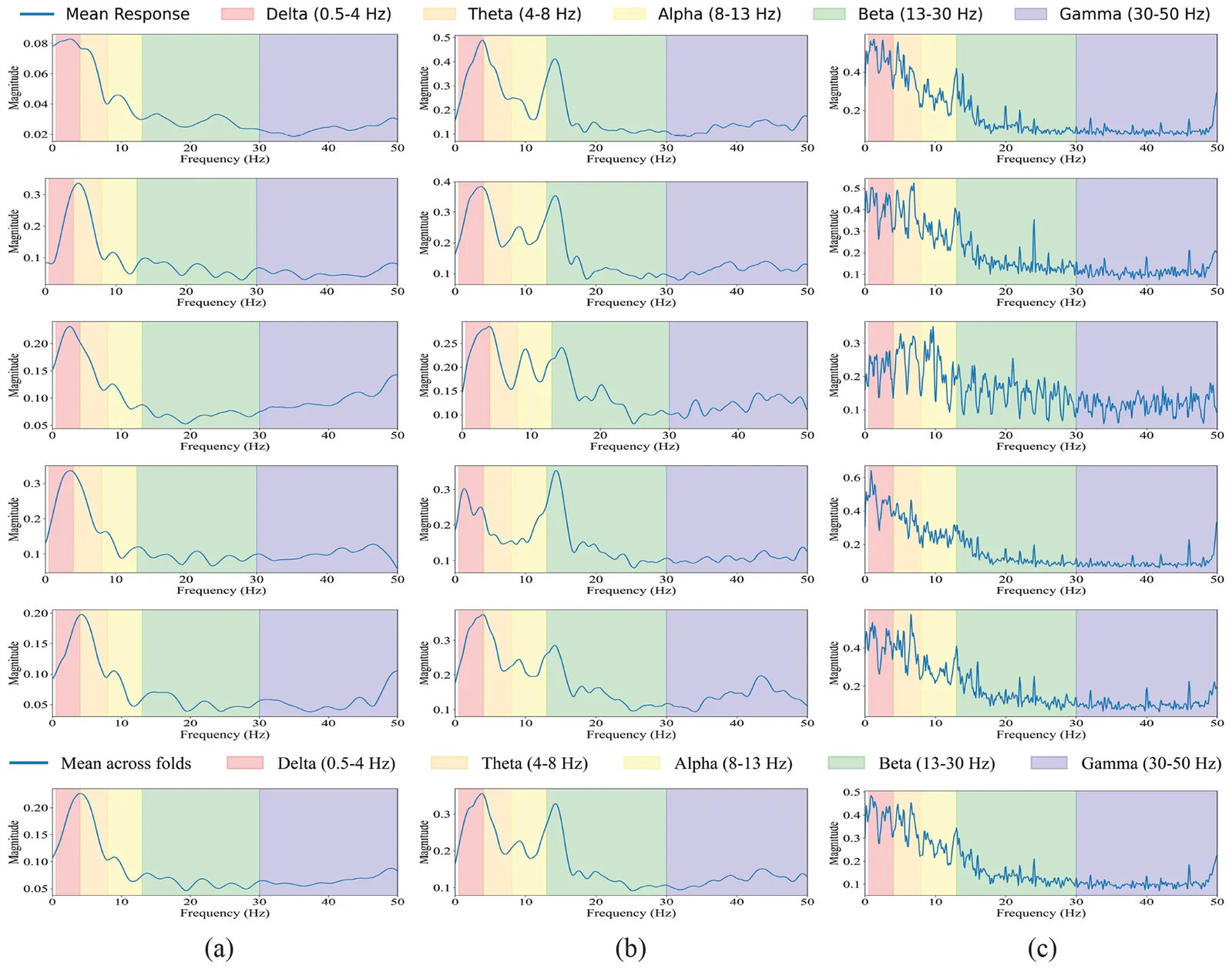
Frequency response of MSFA-CN, **(a)** Slow-wave branch (kernel size = fs/4), **(b)** Two-peak branch(kernel size = fs/2), **(c)** Rhythm coupling branch(kernel size = fs*4).

Specifically, slow-wave branch shows stronger responses in low-frequency bands, mainly concentrated in the δ, θ and α ranges. These bands are closely associated with sleep stage transitions, particularly the δ band, which dominates the N3 stage. Therefore, slow-wave branch is more likely to capture features related to slow-wave activity and contributes significantly to deep sleep stage classification.

Two-peak branch demonstrates strong responses in the δ, θ and β bands. Compared with slow-wave branch, it is more sensitive to higher frequencies, especially the β band. Since β activity is typically related to wakefulness and REM sleep, two-peak branch may play a more critical role in characterizing W and REM stages. This difference in spectral sensitivity suggests that the model structurally assigns distinct rhythmic patterns to different branches.

Due to the use of larger convolution kernels and a deeper receptive field, rhythm coupling branch covers a broader spectral range and shows notable responses across multiple bands, including δ, θ, α and β. This indicates stronger cross-band integration capability, enabling it to capture multi-scale time-frequency information and potential coupling between rhythms, thus providing more global discriminative features.

Overall, the differentiated spectral responses of the three branches validate the rationality of the MSFA-CN design. Convolution kernels at different scales are sensitive to specific frequency bands, and feature fusion enables comprehensive representation of multi-band EEG information. This multi-scale feature extraction mechanism enhances the model's sensitivity to sleep transition boundaries and improves its ability to distinguish between different sleep stages.

### 4.6 Anti-correlation analysis

To test the core hypothesis that inter-band anticorrelation increases during sleep stage transitions, and to provide quantitative support for the physiological design of MSFA-CN, we first performed a systematic analysis of Pearson correlation coefficients among EEG frequency band powers across all sleep stages. In Figure 11, results showed that as sleep deepened from W to N3, the correlations between delta and high-frequency bands (α, β and γ) decreased markedly, approaching zero or turning negative. Specifically, the δ-α correlation dropped from +0.44 in W to +0.09 in N3, and the δ-β correlation decreased from +0.54 to -0.13. These findings quantitatively confirmed the inter-band anticorrelation pattern in deep sleep, characterized by δ dominance and concurrent suppression of α, β, and γ activities. In contrast, during wakefulness, all frequency bands generally exhibited positive correlations, with the β-γ correlation reaching as high as +0.94.

**Figure 11:**
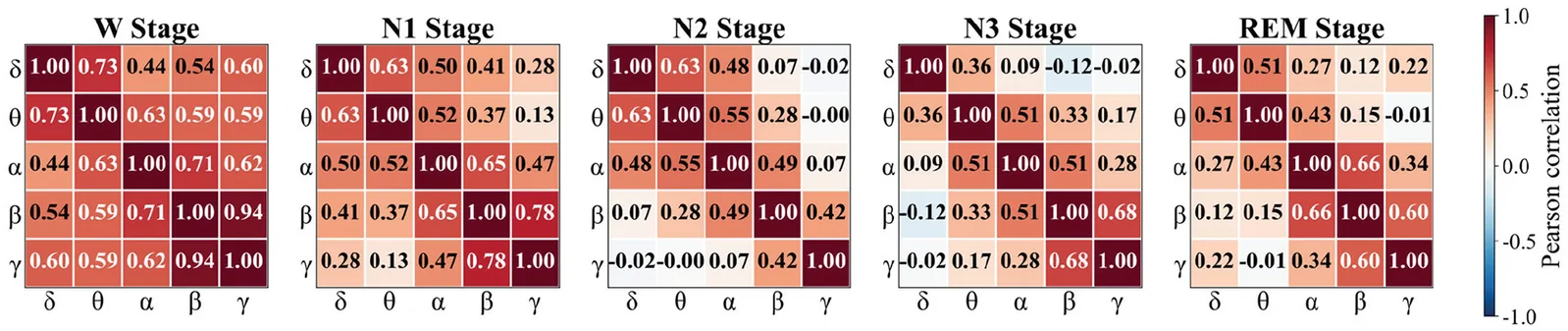
Heatmap of Pearson correlation coefficients among frequency bands across different sleep stages on SleepEDF-20.

Given that W and N3 represent the two extreme states of wakefulness and deep sleep, respectively, and thus best capture the dynamic range of inter-band relationships, in Figure 12, we focused our subsequent analyses on these two stages. The analytical procedure was as follows: (1) For each subject, we computed Pearson correlation coefficients of band powers across all epochs within W and N3 separately. (2) Only subjects with at least 30 epochs available in both stages were included to ensure reliable correlation estimation. (3) For each frequency pair, paired Wilcoxon signed-rank tests were applied to compare W and N3, and the resulting *p*-values were corrected for multiple comparisons across all 10 frequency pairs using the Benjamini-Hochberg false discovery rate (BH-FDR) procedure.

**Figure 12:**
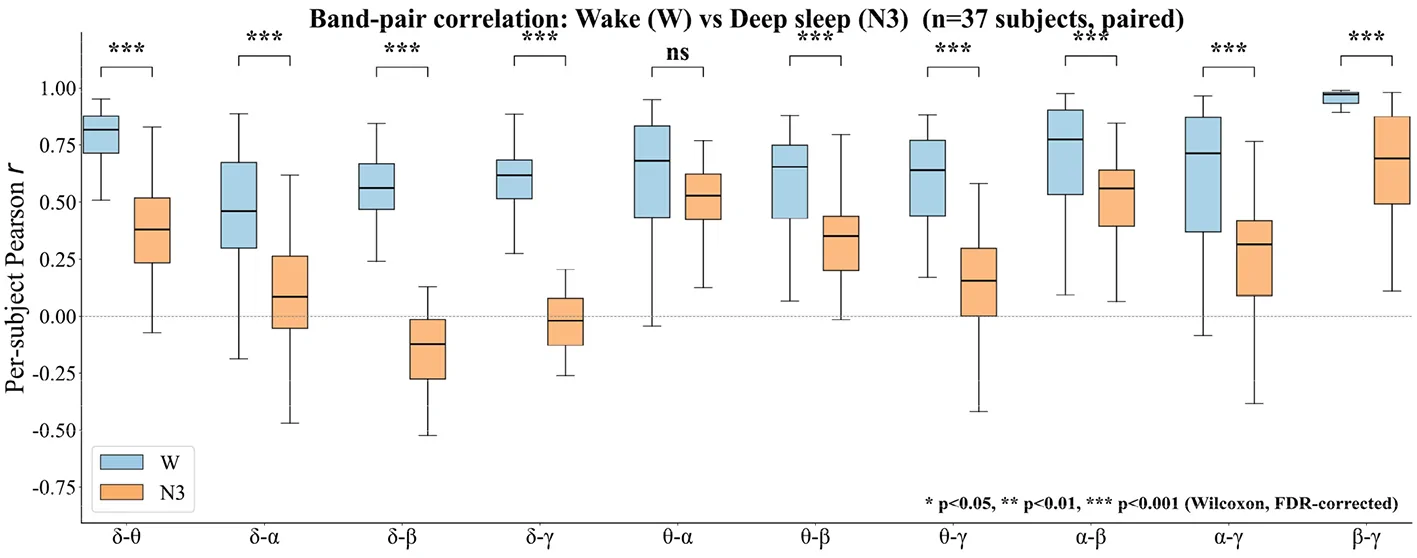
Significance analysis of changes in Pearson correlation coefficients for frequency-band pairs from Wake to N3 on SleepEDF-20 (***p < 0.001, ns: *p* > 0.05).

The results indicated that 9 out of the 10 frequency pairs showed significant differences between W and N3, with only the θ-α pair failing to reach significance. Notably, the frequency pairs involving δ and high-frequency bands exhibited the most pronounced changes: the δ-β correlation dropped significantly from +0.54 in W to -0.12 in N3, and the δ-γ correlation decreased from +0.60 to -0.02. These subject-level paired statistical results rigorously validated the significant inter-band anticorrelation pattern in deep sleep, marked by δ band dominance and concurrent suppression of β and γ activities.

### 4.7 Parameter specificity analysis

MHA and MSFA-CN are the core components of the model, and the selection of their parameters has a significant impact on the model's performance. Therefore, we analyzed the influence of parameters on the network. Regarding the number of heads, the number of heads in MHA should be divisible by the length of the features. Since the feature length of the SleepEDF-20 and SleepEDF-78 datasets is 330, the number of heads is selected from 2, 3, 5, 6, and 10. As for the selection of the dimensionality reduction channel parameter in MSFA-CN, we conducted experiments from 10 to 40. From Figure 13, it can be seen that the performance of SleepEDF-20 fluctuates significantly, which is speculated to be due to the small size of the dataset, making the model sensitive to parameter selection. As the number of heads h increases, the overall performance of the network first increases and then decreases. This is because initially, the increase in the number of heads helps the network find more meaningful features and context relationships.

**Figure 13:**
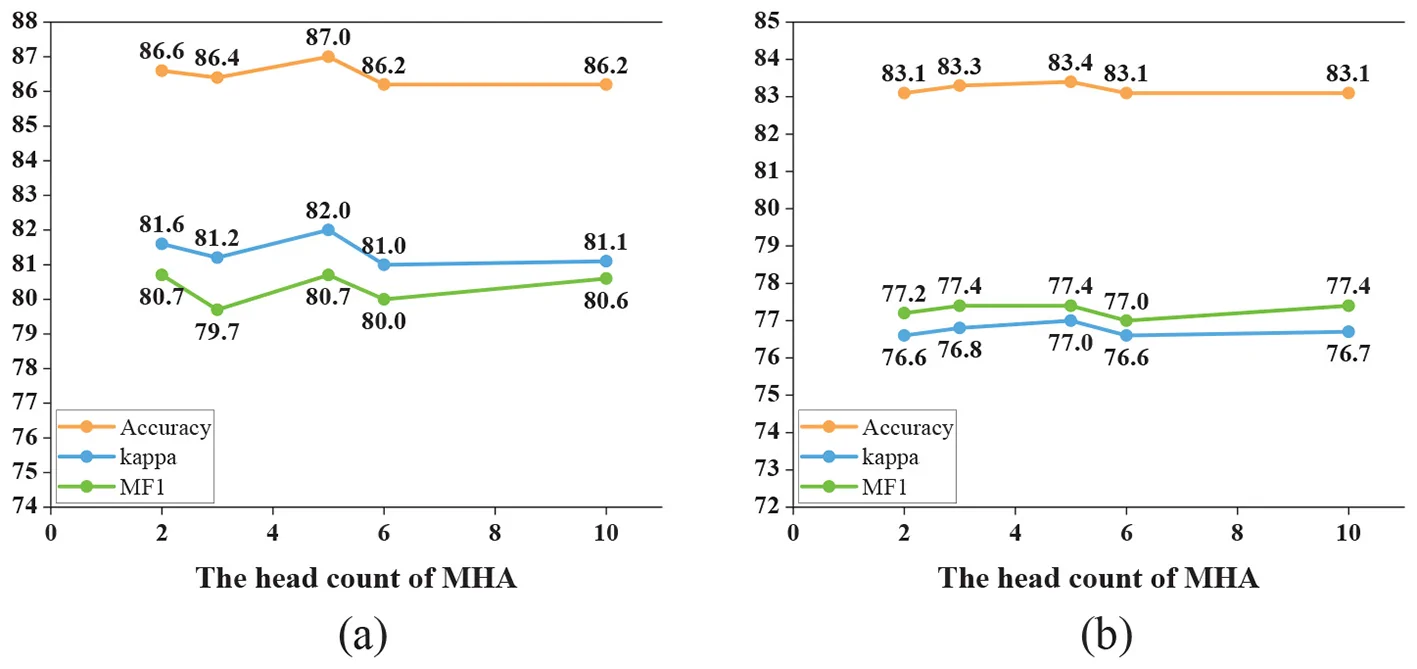
The head count analysis of MHA: **(a)** SleepEDF-20, **(b)** SleepEDF-78.

However, as the number of heads increases, the feature length of each head decreases, making it difficult to capture context relationships, and the performance of the network declines. From the curves in Figure 13, it can be observed that the network performs best when h = 5 on both the SleepEDF-20 and SleepEDF-78 datasets. Therefore, the number of heads h is ultimately set to 5 in the experiment. For the selection of MSFA-CN parameters, in Figure 14, as the number of channels increases, the network improves its accuracy by retaining more features. However, after exceeding 30, the accuracy decreases. This is because too many features can actually affect classification performance. Therefore, the number of channels is ultimately set to 30 in the experiment.

**Figure 14:**
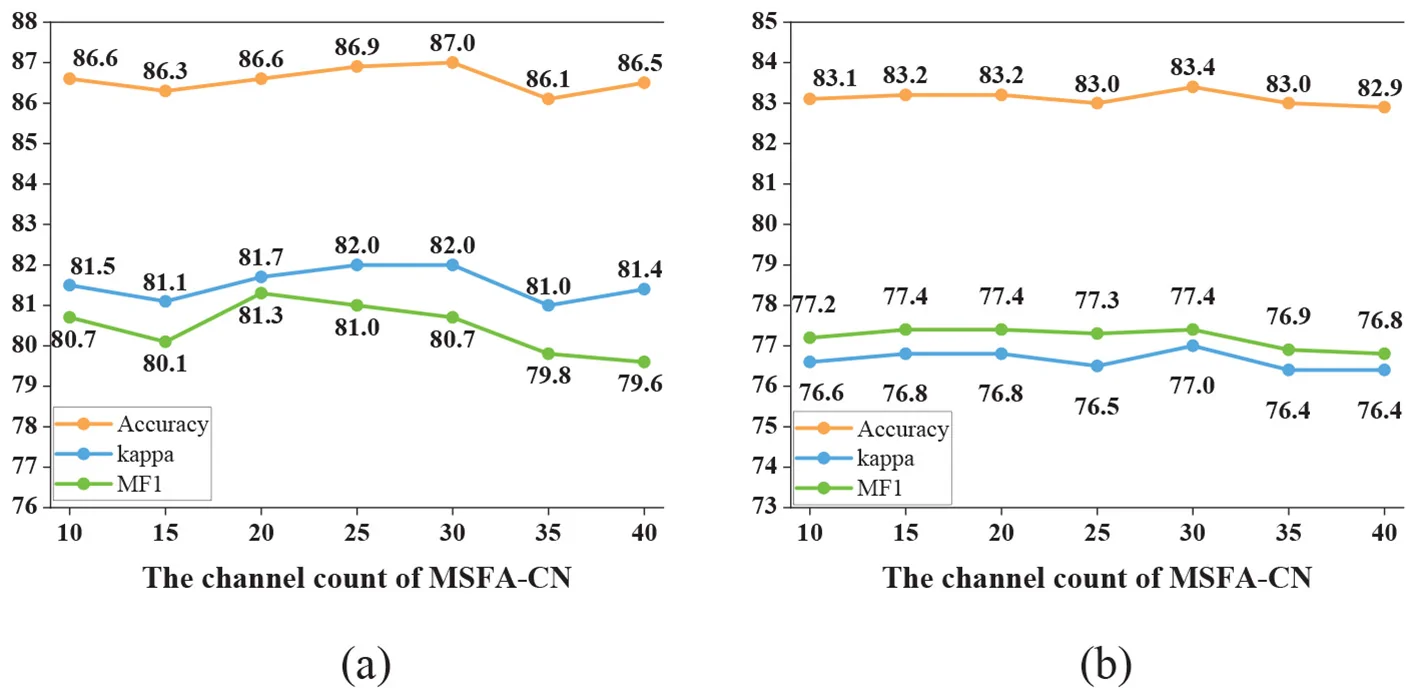
The channel count analysis of MSFA-CN: **(a)** SleepEDF-20, **(b)** SleepEDF-78.

### 4.8 Attention comparison

To demonstrate that the performance improvement of the proposed attention module is not coincidental, we compared three different attention mechanisms: ECA (Efficient Channel Attention) (Wang et al., 2020), AA (Double Attention) (Chen et al., 2018), and SE. Ten repeated experiments were conducted for each mechanism, and the results based on three performance metrics (Accuracy, MF1, and kappa) are presented in Figure 15. The Kruskal-Wallis non-parametric test revealed significant differences among the four attention mechanisms (*p* < 0.05). Further *post-hoc* analysis using Dunn's test showed that, compared with other attention mechanisms, DRA achieved the highest average values across all three metrics with a more concentrated sample distribution, followed by ECA, then SE and AA. This may be attributed to DRA's ability to jointly model temporal variations and inter-channel dependencies, thereby enhancing feature discriminability. ECA also exhibited competitive performance by modeling inter-channel relationships via 1D convolution. In contrast, SE introduced unnecessary information loss due to its two fully connected layers, leading to inferior performance. AA's complex computations adversely affected negative correlations among features, resulting in the poorest performance. These findings confirm that our method not only considers temporal dynamics but also establishes dependencies among feature channels, enabling adaptive feature recalibration. Hence, our model more effectively captures variations in EEG signals, thereby improving overall network performance.

**Figure 15:**
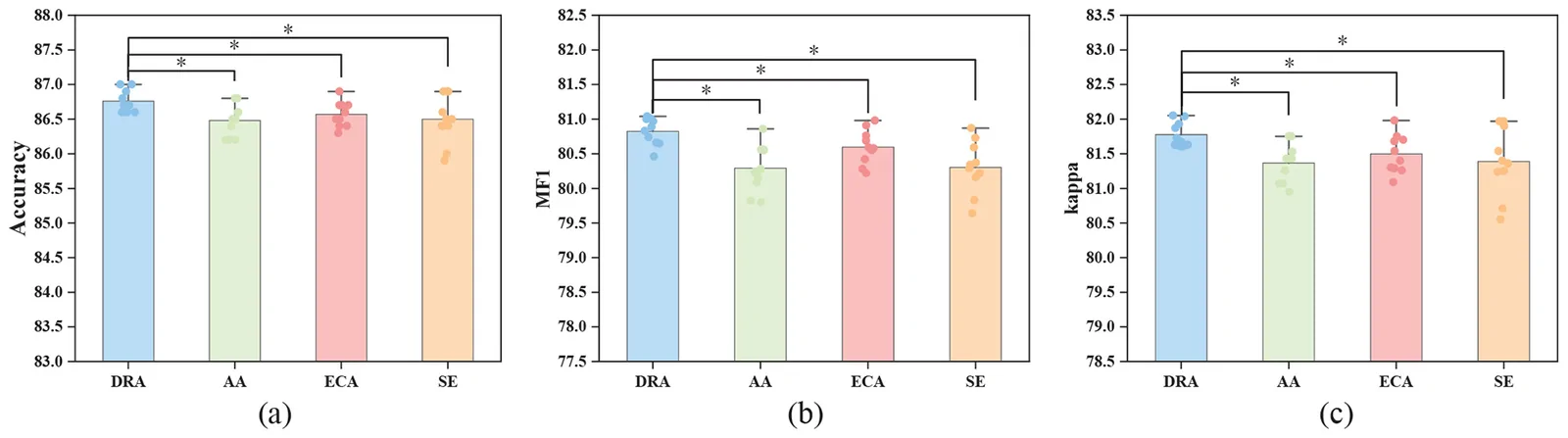
Comparison of different attention modules **(a)** Accuracy, **(b)** MF1, **(c)** kappa. The kruskal-wallis test results for individual sample-wise metrics across three methods were annotated on the graph, *p < 0.05.

### 4.9 Impact of sampling rate

To investigate the impact of sampling rate on the model, we resampled SleepEDF-20 to 50 Hz and 100 Hz and conducted experiments. In Table 4, the accuracy remains stable at 50 Hz and 200 Hz, with notable changes in N1 stage accuracy. At 50 Hz, halving the features causes only a 0.4% drop, because low-frequency information suffices for sleep staging and the doubled receptive field better captures slow waves. However, the lower sampling rate may lose some high-frequency edge information required to distinguish W and N1 stages, reducing N1 accuracy by 5.4% compared to 100 Hz. At 200 Hz, doubling the features leads to a 0.9% drop, likely due to high-frequency noise and the halved receptive field missing key patterns such as K-complexes. Although higher sampling rate provides finer temporal resolution, high-frequency noise may disrupt the high-frequency transient features of the N1 stage, reducing its accuracy by 3.0% compared to 100 Hz. Notably, REM stage accuracy drops by 2.0%, possibly because the model over-focuses on high-frequency pseudo-eye movements introduced by interpolation, misclassifying other stages as REM. In conclusion, the model demonstrates excellent classification performance across EEG inputs with varying sampling rates, exhibiting higher tolerance to downsampling compared to upsampling. Nevertheless, N1 stage accuracy is the most sensitive to sampling rate, and the high-frequency noise from up-sampling also affects the accuracy of N1 and REM stages.

**Table 4:** Performance at different sampling rates on SleepEDF-20.

Sampling rate	Acc	MF1	kappa	W	N1	N2	N3	REM
50 Hz	86.6	80.5	0.82	91.5	52.1	89.5	89.4	82.7
100 Hz	87.0	80.7	0.82	91.5	57.5	89.0	89.5	82.9
200 Hz	86.0	79.3	0.81	90.7	54.5	88.5	88.9	80.9

### 4.10 Computational efficiency analysis

Besides classification performance, computational efficiency and deployment feasibility are important for the practical translation of single-channel EEG sleep staging. Therefore, in Table 5, we compare the proposed model with Zhou et al. (2022) in terms of parameter count, floating point operations (FLOPs), per-sample inference time (MIT), and per-sample memory usage (MU). All measurements were performed on a fixed hardware platform (NVIDIA RTX 3090 GPU). The results show that the proposed model has 1.43M parameters, 0.65G FLOPs, a MIT of 1.997 ± 0.363 ms, and a MU of 11.73 MB. Compared with the baseline model, the parameter count increases slightly. However, FLOPs decrease by about 62.9%, and memory usage decreases by about 77.4%. The inference time also remains at the millisecond level. This indicates that the model has clear advantages in computational complexity and memory overhead. It is therefore more suitable for deployment in resource-constrained scenarios.

**Table 5:** Computational performance of our model.

Model	Params	FLOPs	MIT/ms	MU/MB	TT/s
Zhou et al. (2022)	0.51M	1.75G	1.119 ± 0.443	51.87	-
Ours	1.43M	0.65G	1.997 ± 0.363	11.73	0.3

Params, Parameters; FLOPs, Floating Point Operations; MIT, Model Inference Time/1 sample; MU, Memory Usage/1 sample; TT, Training Time/1 epoch.

Sleep staging typically uses 30-s epochs. The proposed model has a MIT of about 2 ms. This is far shorter than one epoch. Even with preprocessing, channel selection, and post-processing, the total latency remains within seconds. Therefore, the model supports real-time or near-real-time operation. It can be used for online sleep monitoring and continuous sleep staging.

The main constraints of wearable sleep monitors are computing power, memory, and energy consumption, not parameter count alone. The proposed model uses only 11.73 MB of memory per sample and 0.65G FLOPs. It is a lightweight model. It therefore meets the basic conditions for wearable integration. For deployment, model quantization, pruning, and operator optimization can be applied. These techniques help adapt the model to low-power MCUs, mobile NPUs, or edge AI chips. However, the current experiments mainly assess theoretical computational cost and inference time on specific hardware. On-device power consumption and long-term stability are not yet systematically evaluated. These aspects require further verification.

Table 5 shows that the proposed model has good deployment potential in computational efficiency, memory usage, and inference speed. It supports real-time operation, wearable compatibility, and portable home monitoring. These characteristics enhance the translational significance of this study. We will conduct on-device deployment experiments. We will evaluate how quantization, pruning, and power optimization affect model performance and practical operation. This will help promote the application of the model in real sleep monitoring scenarios.

### 4.11 DRA weight visualization analysis

To more intuitively show which temporal regions of the signal the DRA module focuses on when processing features, in Figure 16, we adopted Grad-CAM to generate attention heatmaps overlaid on the original EEG signals. We selected typical samples from each of the five stages and displayed the alignment between the heatmaps and known physiological events in time. In the W stage (16a), the module mainly focuses on one region with obvious fluctuations and three relatively stable segments. This suggests attention to changes in alertness and quiet restfulness. In the N1 stage (16b), the model primarily focuses on several fluctuating regions, reflecting the transition from wakefulness to sleep. In the N2 stage (16c), the model concentrates on regions with pronounced fluctuations, including peaks and troughs. These may correspond to the presence of sleep spindles or K-complexes, which are key hallmarks of this stage. In the N3 stage (16d), the model focuses on several high-amplitude slow-wave regions, which exactly correspond to the key markers of deep sleep. In the REM stage (16e), attention is distributed across a combination of peak regions and stable segments. This may be related to vivid dreaming and heightened brain activity during this stage. These observations confirm that DRA can help the model detect and emphasize the regions in EEG signals that are useful for sleep stage classification.

**Figure 16:**
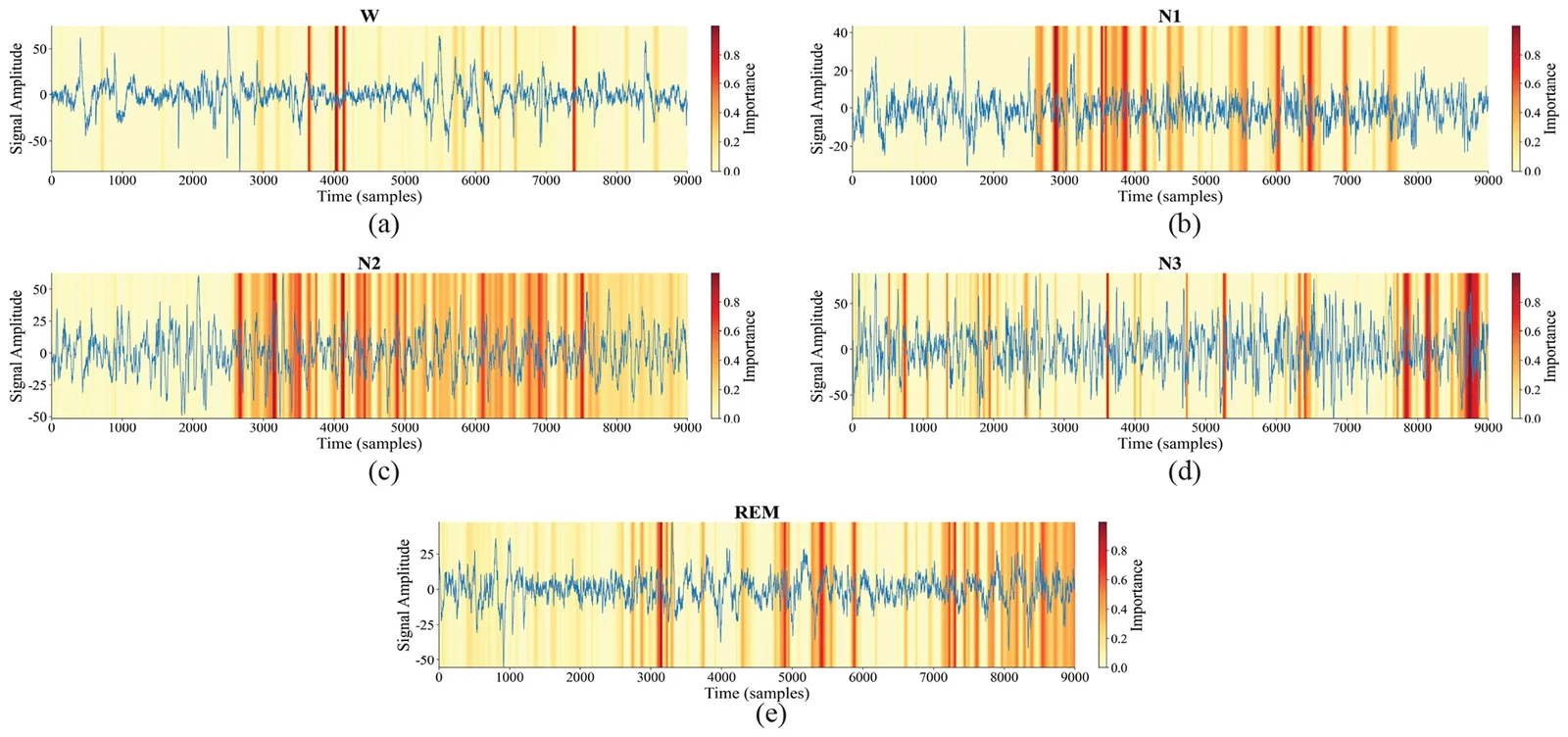
The 90-second attention heatmaps are overlaid for each sleep stage **(a)** Wake stage, **(b)** N1 stage, **(c)** N2 stage, **(d)** N3 stage, **(e)** REM stage.

### 4.12 Noise resistance analysis

To evaluate the model's tolerance to noise, we added a certain level of Gaussian white noise to the original EEG signals in the validation set on SleepEDF-20. To better evaluate the impact of noise on each fold, we compared the average metrics across all 20-folds. In Table 6, it can be observed that before the signal-to-noise ratio (SNR) reaches 20 dB, the model performance shows no significant degradation. At 20 dB, Acc drops by 0.5%, MF1 decreases by 0.4, and kappa decreases by 0.01. When the SNR drops to 10 dB, Acc declines to 67.4%, MF1 falls to 60.5, and kappa decreases to 0.57. Due to the presence of noise, the model's classifications gradually shift toward the Wake and N1 stages. This occurs because the low-frequency components of the noise prevent the model from correctly identifying deep sleep features. When the SNR reaches 0 dB, the noise power is comparable to the signal power. At this point, the model can no longer correctly recognize valid signals. Acc drops significantly by 63.4%, MF1 drops by 66.7, and kappa drops by 0.75. Most samples are misclassified as the Wake stage under this condition. This is because the noise power greatly increases the signal amplitude. The model thus interprets the high-amplitude signal as a characteristic of the Wake stage. It is worth noting that our experiments only discussed the impact of Gaussian white noise on the model. In real-world prediction, EEG interference also includes motion artifacts, which can likewise affect the model's accuracy.

**Table 6:** Performance at different signal-to-noise ratio (SNR) on SleepEDF-20.

SNR	Acc	MF1	kappa	W	N1	N2	N3	REM
100 dB	83.4	76.0	0.77	89.0	37.8	86.3	91.1	82.1
80 dB	83.4	76.0	0.77	89.0	37.8	86.3	91.1	82.1
60 dB	83.4	76.0	0.77	89.0	37.9	86.3	91.1	82.1
40 dB	83.4	76.0	0.77	89.0	37.9	86.3	91.1	82.2
20 dB	82.9	75.6	0.76	90.1	39.0	85.1	91.7	80.9
10 dB	67.4	60.5	0.57	94.6	40.8	66.9	52.2	63.3
0 dB	20.0	9.3	0.01	99.1	9.7	0.1	2.0	0.2

### 4.13 Importance analysis of causal convolution

The above ablation experiments have already verified the effectiveness of each major module. However, the specific contribution of the causal convolution design within the CTN module still requires further analysis. The CTN module employs causal convolution to generate the query, key, and value matrices. The core motivation is to make the CTN satisfy temporal causal constraints and prevent it from leveraging future information to perceive transition rules during training. This ensures that the model can achieve better generalization performance. To demonstrate this, we designed a set of controlled ablation experiments. We replaced the causal convolution in the CTN module with standard convolution, while keeping all other network structures, training strategies, and hyperparameters unchanged. By comparing the classification performance under the two settings, we can quantitatively evaluate the actual effect of the causal design in preventing information leakage and improving generalization ability. In Table 7, the performance of standard 1D convolution is slightly lower than that of causal convolution across all metrics. This is because traditional 1D convolution neglects the temporal order constraints within the features, leading to future information leakage. In contrast, causal convolution enforces temporal constraints on the features, which enables the CTN to better learn sleep transition rules and thus improves performance.

**Table 7:** Performance at different convolution on SleepEDF-20.

Model	Acc	MF1	kappa	W	N1	N2	N3	REM
1D convolution	86.2	80.3	0.81	90.8	57.4	88.9	88.9	81.0
causal convolution	87.0	80.7	0.82	91.5	57.5	89.0	89.5	82.9

## 5 Discussion

Although our model has achieved good results in automatic sleep stage classification, this study also has certain limitations. First, due to ethical restrictions on human data, we only validated the model on publicly available and legally accessible datasets. There is a lack of validation on external clinical datasets. Second, although we have improved the classification accuracy of the N1 stage to 57.5%, this may depend on the contextual input. In addition, the imbalanced sample distribution in sleep datasets is another factor affecting model accuracy.

In future research, we believe that further exploration can be pursued in three directions: multi-channel input, improving N1 stage classification accuracy, and addressing the sample imbalance problem. Regarding the improvement of N1 stage classification accuracy, the main factor affecting the overall accuracy of automatic sleep stage classification is the classification accuracy of the N1 stage. Future research can focus on this aspect by designing specific architectures and developing dedicated loss functions to improve N1 classification accuracy. As for the sample imbalance problem, future research can use specific techniques such as data augmentation to alleviate this issue and balance the sample sizes. In addition, finding larger databases to train the model may also be beneficial.

## 6 Conclusion

In this study, we propose a sleep stage classification framework that leverages single-channel EEG signals. Initially, the model extracts distinct frequency band features using MSFA-CN, followed by DRA for feature fusion across temporal and channel dimensions. Subsequently, a CTN integrated with MHA is employed to discern the transition patterns of sleep stages. To counteract data imbalance challenges, we incorporate a focal loss function. Comprehensive experiments on three public datasets reveal that our proposed model surpasses current state-of-the-art techniques in all evaluative metrics. Furthermore, ablation studies affirm the efficacy of each module from both qualitative and quantitative standpoints.

## Data Availability

The original contributions presented in the study are included in the article/supplementary material, further inquiries can be directed to the corresponding author.

## References

[B1] Andrillon T. Nir Y. Staba R. J. Ferrarelli F. Fried I. (2011). Sleep spindles in humans: insights from intracranial eeg and unit recordings. J. Neurosci. 31, 17821–17834. doi: 10.1523/JNEUROSCI.2604-11.2011 22159098 PMC3270580

[B2] Ba J. L. Kiros J. R. Hinton G. E. (2016). Layer normalization. arXiv [preprint]. arXiv:1607.06450.

[B3] Berry R. B. Brooks R. Gamaldo C. Harding S. M. Lloyd R. M. Quan S. F. . (2017). AASM scoring manual updates for 2017 (version 2.4). J. Clin. Sleep Med. 13, 665–666. doi: 10.5664/jcsm.6576 28416048 PMC5406946

[B4] Cao Y. Xiang W. Wei J. Cao S. Tian X. Zhong J. . (2026). Crossfusionsleepnet: A multimodal deep learning model for automatic sleep stage classification. Biomed. Sig. Process. Control 112:108538. doi: 10.1016/j.bspc.2025.108538

[B5] Chambon S. Galtier M. N. Arnal P. J. Wainrib G. Gramfort A. (2018). A deep learning architecture for temporal sleep stage classification using multivariate and multimodal time series. IEEE Trans. Neural Syst. Rehabil. Eng. 26, 758–769. doi: 10.1109/TNSRE.2018.2813138 29641380

[B6] Chen Y. Kalantidis Y. Li J. Yan S. Feng J. (2018). A^2^-nets: double attention networks. arXiv [preprint].arXiv:1810.11579v1.

[B7] Chen Z. Wu M. Cui W. Liu C. Li X. (2020). An attention based cnn-lstm approach for sleep-wake detection with heterogeneous sensors. IEEE J. Biomed. Health Informat. 25, 3270–3277. doi: 10.1109/JBHI.2020.3006145 32749983

[B8] Cong Z. Zhao M. Gao H. Lou M. Zheng G. Wang Z. . (2025). BiTs-sleepNet: an attention-based two stage temporal-spectral fusion model for sleep staging with single-channel EEG. IEEE J. Biomed. Health Informat. 29, 3366–3376. doi: 10.1109/JBHI.2024.3523908 40030778

[B9] Cui H. Li C. Liu A. Qian R. Chen X. (2023). A dual-branch interactive fusion network to remove artifacts from single-channel EEG. IEEE Trans. Instrument. Meas. 73, 1–12. doi: 10.1109/TIM.2023.3342863

[B10] Dai W. Dai C. Qu S. Li J. Das S. (2017). “Very deep convolutional neural networks for raw waveforms,” in Proceedings of IEEE International Conference on Acoustics, Speech and Signal Processing (ICASSP) (New York, NY: ACM), 421–425. doi: 10.1109/ICASSP.2017.7952190

[B11] Eldele E. Chen Z. Liu C. Wu M. Kwoh C.-K. Li X. . (2021). An attention-based deep learning approach for sleep stage classification with single-channel EEG. IEEE Trans. Neural Syst. Rehabil. Eng. 29, 809–818. doi: 10.1109/TNSRE.2021.3076234 33909566

[B12] Freeman D. Sheaves B. Waite F. Harvey A. G. Harrison P. J. (2020). Sleep disturbance and psychiatric disorders. Lancet Psych. 7, 628–637. doi: 10.1016/S2215-0366(20)30136-X 32563308

[B13] Fu G. Zhou Y. Gong P. Wang P. Shao W. Zhang D. (2023). A temporal-spectral fused and attention-based deep model for automatic sleep staging. IEEE Trans. Neural Syst. Rehabil. Eng. 31, 1008–1018. doi: 10.1109/TNSRE.2023.3238852 37022069

[B14] Goldberger A. L. Amaral L. A. Glass L. Hausdorff J. M. Ivanov P. C. Mark R. G. . (2000). Physiobank, physiotoolkit, and physioNet: components of a new research resource for complex physiologic signals. Circulation 101, e215–e220. doi: 10.1161/01.CIR.101.23.e215 10851218

[B15] Grandner M. A. (2020). Sleep, health, and society. Sleep Med. Clin. 15, 319–340. doi: 10.1016/j.jsmc.2020.02.017 32386705

[B16] Hassan A. R. Bhuiyan M. I. H. (2016). Automatic sleep scoring using statistical features in the emd domain and ensemble methods. Biocybern. Biomed. Eng. 36, 248–255. doi: 10.1016/j.bbe.2015.11.001

[B17] Hinton G. Roweis S. (2003). Stochastic Neighbor Embedding. Cambridge, MA: MIT Press.

[B18] Hirshkowitz M. (2016). Polysomnography challenges. Sleep Med. Clin. 11, 403–411. doi: 10.1016/j.jsmc.2016.07.002 28118865

[B19] Hu J. Shen L. Sun G. (2018). “Squeeze-and-excitation networks,” in Proceedings of the IEEE Conference on Computer Vision and Pattern Recognition (Salt Lake City, UT: IEEE), 7132–7141. doi: 10.1109/CVPR.2018.00745

[B20] Hu Y. Yang X. Xu Y. Sun J. (2025). Causal-aware reliability assessment of single-channel eeg for transformer-based sleep staging. Front. Neurosci. 19:1670124. doi: 10.3389/fnins.2025.1670124 41132593 PMC12540495

[B21] Huang L. Zhang W. Li C. Zhao W. Peng H. Chen X. (2025). EEGCMCNet: a hybrid network for sleep stage classification. IEEE Sens. J. 25, 25324–25337. doi: 10.1109/JSEN.2025.3574141

[B22] Huang W. Cheng J. Yang Y. Guo G. (2019). An improved deep convolutional neural network with multi-scale information for bearing fault diagnosis. Neurocomputing 359, 77–92. doi: 10.1016/j.neucom.2019.05.052

[B23] Kohen J. (1960). A coefficient of agreement for nominal scale. Educ. Psychol. Meas. 20, 37–46. doi: 10.1177/001316446002000104

[B24] Kwon H. B. Choi S. H. Lee D. Son D. Yoon H. Lee M. H. . (2021). Attention-based LSTM for non-contact sleep stage classification using IR-UWB radar. IEEE J. Biomed. Health Informat. 25, 3844–3853. doi: 10.1109/JBHI.2021.3072644 33848253

[B25] Lin A. Liu K. K. Bartsch R. P. Ivanov P. C. (2020a). Dynamic network interactions among distinct brain rhythms as a hallmark of physiologic state and function. Commun. Biol. 3:197. doi: 10.1038/s42003-020-0878-4 32341420 PMC7184753

[B26] Lin T.-Y. Goyal P. Girshick R. He K. Dollàr P. (2020b). Focal loss for dense object detection. IEEE Trans. Pattern Anal. Mach. Intell. 42, 318–327. doi: 10.1109/TPAMI.2018.2858826 30040631

[B27] Luyster F. S. Strollo Jr P. J. Zee P. C. Walsh J. K. (2012). Sleep: a health imperative. Sleep 35, 727–734. doi: 10.5665/sleep.1846 22654183 PMC3353049

[B28] Ma J. Ren Z. Zhang T. Ding Y. Lu Y. Peng Y. (2025). Transformer-based contrastive learning method for automated sleep stages classification. J. Shanghai Jiaotong Univ. 30, 720–732. doi: 10.1007/s12204-024-2734-z

[B29] Madhuchhanda B. Diptadip M. Debasis C. (2026a). EEG-driven pain intensity estimation using feature-level fusion and deep ensemble models. Neural Comput. Appl. 38:627. doi: 10.1007/s00521-026-12355-x

[B30] Madhuchhanda B. Diptadip M. Debasis C. (2026b). Parallel multi-stage artificial neural network and lstm for precise sleep stage recognition. Recent Adv. Comput. Sci. Commun. 19, 1–16. doi: 10.2174/0126662558441354260130173546

[B31] Mourtazaev M. Kemp B. Zwinderman A. Kamphuisen H. (1995). Age and gender affect different characteristics of slow waves in the sleep eeg. Sleep 18, 557–564. doi: 10.1093/sleep/18.7.557 8552926

[B32] Mousavi S. Afghah F. Acharya U. R. (2019). SleepEEGNet: automated sleep stage scoring with sequence to sequence deep learning approach. PLoS ONE 14:e0216456. doi: 10.1371/journal.pone.0216456 31063501 PMC6504038

[B33] Palagini L. Geoffroy P. A. Miniati M. Perugi G. Biggio G. Marazziti D. . (2022). Insomnia, sleep loss, and circadian sleep disturbances in mood disorders: a pathway toward neurodegeneration and neuroprogression? a theoretical review. CNS Spect. 27, 298–308. doi: 10.1017/S1092852921000018 33427150

[B34] Quan S. F. Howard B. V. Iber C. Kiley J. P. Nieto F. J. O'Connor G. T. . (1997). The sleep heart health study: design, rationale, and methods. Sleep 20, 1077–1085. doi: 10.1093/sleep/20.12.1077 9493915

[B35] Rahman M. M. Bhuiyan M. I. H. Hassan A. R. (2018). Sleep stage classification using single-channel EOG. Comput. Biol. Med. 102, 211–220. doi: 10.1016/j.compbiomed.2018.08.022 30170769

[B36] Ramar K. Malhotra R. K. Carden K. A. Martin J. L. Abbasi-Feinberg F. Aurora R. N. . (2021). Sleep is essential to health: an american academy of sleep medicine position statement. J. Clin. Sleep Med. 17, 2115–2119. doi: 10.5664/jcsm.9476 34170250 PMC8494094

[B37] Ren Z. Ma J. Ding Y. (2025). FlexibleSleepNet: a model for automatic sleep stage classification based on multi-channel polysomnography. IEEE J. Biomed. Health Informat. 29, 3488–3501. doi: 10.1109/JBHI.2025.3525626 40030855

[B38] Shen Q. Xin J. Liu X. Wang Z. Li C. Huang Z. . (2023). LGSleepNet: an automatic sleep staging model based on local and global representation learning. IEEE Trans. Instrument. Meas. 72, 1–14. doi: 10.1109/TIM.2023.3298639

[B39] Sun J. Chen C. Ding W. Hu X. (2026). Sleeplog: local-global deep fusion learning for sleep staging transformer. IEEE Trans. Artif. Intell. 7, 1084–1096. doi: 10.1109/TAI.2025.3591587

[B40] Sun J. Wang R. Zhao G. Chen C. Qu Y. Li J. . (2023). “START: automatic sleep staging with attention-based cross-modal learning transformer,” in Proceedings of IEEE International Conference on Bioinformatics and Biomedicine (BIBM) (Istanbul: IEEE), 1421–1428. doi: 10.1109/BIBM58861.2023.10385393

[B41] Supratak A. Dong H. Wu C. Guo Y. (2017). DeepSleepNet: a model for automatic sleep stage scoring based on raw single-channel EEG. IEEE Trans. Neural Syst. Rehabil. Eng. 25, 1998–2008. doi: 10.1109/TNSRE.2017.2721116 28678710

[B42] Vaswani A. Shazeer N. Parmar N. Uszkoreit J. Jones L. Gomez A. N. . (2017). Attention is all you need. Adv. Neural Inf. Process. Syst. 30, 6000–6010. doi: 10.5555/3295222.3295349

[B43] Vaughn B. V. Giallanza P. (2008). Technical review of polysomnography. Chest 134, 1310–1319. doi: 10.1378/chest.08-0812 19059962

[B44] Wang J. Wang X. Ning X. Lin Y. Phan H. Jia Z. (2025). Subject-adaptation salient wave detection network for multimodal sleep stage classification. IEEE J. Biomed. Health Informat. 29, 2172–2184. doi: 10.1109/JBHI.2024.3512584 40030418

[B45] Wang Q. Wu B. Zhu P. Li P. Zuo W. Hu Q. (2020). “ECA-Net: efficient channel attention for deep convolutional neural networks,” in Proceedings of IEEE/CVF Conference on Computer Vision and Pattern Recognition (CVPR) (Seattle, WA: IEEE), 11531–11539. doi: 10.1109/CVPR42600.2020.01155

[B46] Wei Y. Zhu Y. Zhou Y. Yu X. Luo Y. (2023). Automatic sleep staging based on contextual scalograms and attention convolution neural network using single-channel EEG. IEEE J. Biomed. Health Informat. 28, 801–811. doi: 10.1109/JBHI.2023.3332503 37955995

[B47] Zaman A. Kumar S. Shatabda S. Dehzangi I. Sharma A. (2024). SleepBoost: a multi-level tree-based ensemble model for automatic sleep stage classification. Med. Biol. Eng. Comput. 62, 2769–2783. doi: 10.1007/s11517-024-03096-x 38700613

[B48] Zhang G.-Q. Cui L. Mueller R. Tao S. Kim M. Rueschman M. . (2018). The national sleep research resource: towards a sleep data commons. J. Am. Med. Informa. Assoc. 25, 1351–1358. doi: 10.1093/jamia/ocy064 29860441 PMC6188513

[B49] Zhang W. Li C. Peng H. Qiao H. Chen X. (2024). CTCNet: a CNN transformer capsule network for sleep stage classification. Measurement 226:114157. doi: 10.1016/j.measurement.2024.114157

[B50] Zhao C. Li J. Guo Y. (2022). SleepContextNet: a temporal context network for automatic sleep staging based single-channel EEG. Comput. Methods Program. Biomed. 220:106806. doi: 10.1016/j.cmpb.2022.106806 35461126

[B51] Zhou D. Wang J. Hu G. Zhang J. Li F. Yan R. . (2022). SingleChannelNet: a model for automatic sleep stage classification with raw single-channel EEG. Biomed. Signal Process. Control 75:103592. doi: 10.1016/j.bspc.2022.103592

[B52] Zhou D. Xu Q. Zhang J. Wu L. Xu H. Kettunen L. . (2024). Interpretable sleep stage classification based on layer-wise relevance propagation. IEEE Trans. Instrument. Meas. 73, 1–10. doi: 10.1109/TIM.2024.3370799

